# LEDT and Idebenone treatment modulate autophagy and improve regenerative capacity in the dystrophic muscle through an AMPK-pathway

**DOI:** 10.1371/journal.pone.0300006

**Published:** 2024-03-18

**Authors:** Heloina Nathalliê Mariano da Silva, Evelyn Mendes Fernandes, Valéria Andrade Pereira, Daniela Sayuri Mizobuti, Caroline Covatti, Guilherme Luiz da Rocha, Elaine Minatel

**Affiliations:** Department of Structural and Functional Biology, Institute of Biology, University of Campinas, Campinas, Brazil; University of Minnesota Medical School, UNITED STATES

## Abstract

**Purpose:**

Considering the difficulties and challenges in Duchenne muscular dystrophy (DMD) treatment, such as the adverse effects of glucocorticoids, which are the main medical prescription used by dystrophic patients, new treatment concepts for dystrophic therapy are very necessary. Thus, in this study, we explore the effects of photobiomodulation (PBM; a non-invasive therapy) and Idebenone (IDE) treatment (a potent antioxidant), applied alone or in association, in dystrophic muscle cells and the quadriceps muscle, with special focus on autophagy and regenerative pathways.

**Methods:**

For the *in vitro* studies, the dystrophic primary muscle cells received 0.5J LEDT and 0.06μM IDE; and for the *in vivo* studies, the dystrophic quadriceps muscle received 3J LEDT and the mdx mice were treated with 200mg/kg IDE.

**Results:**

LEDT and IDE treatment modulate autophagy by increasing autophagy markers (SQSTM1/p62, Beclin and Parkin) and signaling pathways (AMPK and TGF-β). Concomitantly, the treatments prevented muscle degeneration by reducing the number of IgG-positive fibers and the fibers with a central nucleus; decreasing the fibrotic area; up-regulating the myogenin and MCH-slow levels; and down-regulating the MyoD and MHC-fast levels.

**Conclusion:**

These results suggest that LEDT and IDE treatments enhance autophagy and prevented muscle degeneration in the dystrophic muscle of the experimental model. These findings illustrate the potential efficacy of LEDT and IDE treatment as an alternative therapy focused on muscle recovery in the dystrophic patient.

## Introduction

Duchenne muscular dystrophy (DMD), a disease resulting in progressive muscle weakness, is caused by mutation in the dystrophin protein gene [1–3]. Dystrophin mutations trigger a complex cascade of events, such as a significant change in the functioning of the ion channels of the sarcolemma and intracellular organelles and, above all, the sarcoplasmic reticulum and mitochondria regulating ion homeostasis, which is necessary for the correct excitation and relaxation of muscles; exacerbated inflammatory response; and cellular necrosis [4, 5].

Most DMD studies have been carried out in experimental models with mutations in dystrophin, such as the mdx mice. This experimental model has a natural mutation point in exon 23 of the *dmd* gene and an absence of the full-length dystrophin Dp427 protein isoform [6]. However, despite the absence of dystrophin in skeletal and cardiac muscles, adult mdx mice do not exhibit the pathogenic progression characteristic of the dystrophic patient. Severe muscle weakness and accumulation of fat and fibrosis do not appear significantly until almost two years of age [7]. Although the mdx mice presents a milder pathology, this model has been widely used to study the pathways related to the dystrophic phenotype.

Among the dystrophic pathways currently researched, several studies have indicated that the dysregulation of the autophagic flux contributes towards muscle weakness and wasting in the dystrophic muscle [8–11].

Some studies suggest that the autophagic flux is insufficient in the dystrophic muscles and the restoration of this flux recovers muscle function and decreases muscle damage [8–11]. Furthermore, it was reported that the activation of 5’-adenosine monophosphate-activated protein kinase (AMPK) triggered autophagy in the mdx mice, the experimental model of DMD [6], leading to the elimination of damaged mitochondria and the improvement of the diaphragm muscle function [9]. However, there are controversies regarding autophagic flux in DMD, as a previous study has also shown autophagy activation during muscle regeneration at early stages of DMD progression in mdx mice and in dystrophic patients [12]. It has also been reported that both deficient and excessive autophagy result in a pathological cascade and lead to muscular weakness and atrophy symptoms [13]. Increased autophagy can reduce the proliferative capacity of muscle stem cells (MuSCs), which play an important role in the early regeneration of the damaged skeletal muscle. However, the inhibition of autophagy, by the increase of the mammalian target of rapamycin complex 1 (mTORC1) levels, can reverse the defective proliferation of MuSCs [14].

Considering the difficulties and challenges in DMD treatment, such as the adverse effects of glucocorticoids (the main medical prescription used by dystrophic patients [15], new treatment concepts for dystrophic therapy are very necessary. In this respect, two interventions are interesting: the application of photobiomodulation (PBM) and Idebenone (IDE) treatment. PBM is a light-induced therapy that can be performed using laser and/or light emitting diodes (LEDs), over a range of wavelengths and doses [16]. PBM wavelengths between 500 and 700 nm are typically used for the treatment of superficial tissues, such as skin lesions [17]. While wavelengths between 800 and 1000 nm are more used for the treatment of deeper tissues, such as muscle tissue [17]. PBM has been studied in several experimental models, presenting effects that are reported in autophagy modulation [18] and myogenic regulatory factor (MRFs) regulation [19]. PBM, in dystrophic muscles, showed improvement of the regenerative capacity, and decreased inflammatory process and oxidative stress [20, 21]. Regarding IDE, our research group has recently verified that this antioxidant can act as a protective agent against oxidative stress and related signaling mechanisms involved in dystrophic muscle cells [22]. In addition, a previous study reported that IDE improved motor dysfunction by regulating mitophagy in MPTP-treated mice [23]. Reinforcing the potential effect of treatment in conjunction with PBM and IDE, recently, our research group also observed the effects of this combination on calcium and mitochondrial signaling pathways, which were associated with improvement in the dystrophic phenotype [24].

Thus, the purpose of this study is to explore the effects of PBM (a non-invasive therapy) and IDE treatment (a potent antioxidant), applied alone or in association, in dystrophic muscle cells and in the quadriceps muscle, with a special focus on autophagy and signaling pathways.

## Materials and methods

### Animals

All experimental procedures with C57BL/10 (C57BL/10ScCr/PasUnib) and *mdx* (C57BL/10-Dmdmdx/PasUnib) mice were approved by the Ethics Committee in Animal Experimentation of UNICAMP (CEUA #5603-1/2020) and in full compliance with the Brazilian College of Animal Experimentation.

### Experimental design

#### *In vitro* studies

Primary skeletal muscle cells were developed from limb muscles of 28-day-old mice, based on the protocol previously described [25–27]. At least, three individual primary skeletal muscle cell cultures were applied in all experiments.

Four experimental groups were conceptualized: mdxC (untreated dystrophic muscle cells; group used as control); mdxL (dystrophic muscle cells treated with LEDT); mdxI (dystrophic muscle cells treated with IDE); and mdxI+L (dystrophic muscle cells treated with IDE+LEDT).

The muscle cells received IDE at dose of 0.06μM diluted in 0.5% carboxymethylcellulose sodium salt (CMC, Fluka, Buchs, Switzerland) in water and were evaluated after 48 hours of treatment [24].

LEDT (parameters described in Table 1) protocol was based on previous study [28]. The LEDT was applied to primary muscle cells at a perpendicular angle, by a single application, and irradiation was performed by one point at the center of each culture well. LEDT was applied at a distance of 6cm from the bottom plate well. The cells were irradiated inside a laminar flow in a dark room. After LEDT, skeletal muscle cells were maintained in serum-free FM for 48 h. Control cells were not irradiated.

**Table 1:** LEDT parameters.

Device Information		
ThorLabs Mounted High-Power equipment		
	***In vitro* Parameters**	***In vivo* Parameters**
Wavelength (nm)	850	850
Output power (mW)	38	300
Spot size (cm^2^)	9.5	0.32
Power density (mW/cm^2^)	4	937
Energy per point (J)	0.5	3
Energy Density (J/cm^2^)	0.005	9.37
Time per point (s)	13.1	10
Beam shape	Circular	Circular
Operating mode	Continuos	Continuos
Irradiation frequency	1	6, three times per week (for two consecutive weeks)
Irradiation technique	Irradiated once in the center of each primary skeletal muscle cells culture well	Irradiation took place transcutaneously in the center of dystrophic quadriceps muscular venter

#### *In vivo* studies

The animals (14 days old) were divided into five experimental groups: Ctrl group (normal untreated C57BL/10 mice); mdxS (dystrophic mice received sham LEDT and carboxymethylcellulose sodium salt diluted in water); mdxI (dystrophic mice treated with 200mg/kg of IDE diluted in carboxymethylcellulose sodium salt); mdxL (dystrophic mice treated with LEDT-850 nm); and mdxL+I (dystrophic mice treated with LEDT + IDE). The IDE treatment lasted 14 days and mouse were weighed daily till the end of the treatment to regulate the drug dose. Regarding LEDT, the irradiation took place transcutaneously at one point in the center of the dystrophic quadriceps muscular venter, at a distance of 3 cm (without contact). Both hind limbs were irradiated and the application lasted 10 s per session with fixed parameters as described in Table 1. LEDT sham had no energy (0 J) and no power (0 mW) applied over the quadriceps muscular venter. The irradiation occurred three times per week for two consecutive weeks. After the treatments (IDE, LEDT and/or LEDT + IDE), the animals were euthanized with an intraperitoneal injection of a mixture of ketamine hydrochloride (130 mg/kg; Francotar; Virbac) and xylazine hydrochloride (6.8 mg/kg; 2% Virbaxil; Virbac), and the samples (blood and quadriceps muscle) were collected and stored at a low temperature (-80°C) for further tests.

#### Muscle cells diameter

Primary muscle cells were photographed under an inverted microscope (Nikon, Eclipse TS100/TS100F) at 20x magnification. Muscle cell diameters were determined following the protocol of a previous study [27]. The average diameter of each primary muscle cell was calculated as the mean of 10 different muscle cell measurements in 10 different fields (n = 100) using Image-Pro Plus 6.0 software (Media Cybernetic, Silver Spring, MD, USA). The measurements were carried out by a researcher blinded to the origin of the experimental groups. All data were expressed as the mean ± SD.

### Grip strength evaluation

Five measures were obtained for each animal, at the start and end of treatment, for forelimb muscle strength analysis, following the protocol of previous study [29]. Absolute strength was normalized to body weight at 14 and 28 days.

### Creatine Kinase (CK) dosage

The CK (serum and release from quadriceps muscles) protocol was developed based on a previous study [30]. The commercial kit (CK Cinético Crystal, BioClin, Ireland) was used. It is important to highlight that although the quantification of serum CK level is widely used as a biomarker for detecting muscle injuries, the assay can be influenced by age, physical activity and/or pharmacological treatments [31].

### Histopathological analysis

Cryosection of quadriceps muscles were obtained using the cryostat (Leica CM1860-UV) and were used in all histomorphological studies and analyzed using optical light and/or fluorescence microscope. The quantification was obtained using the Image-Pro Express software (Media Cybernetic, Silver Spring, MD, United States) and the results were express as mean ± SD.

### Degenerated muscle fibers

The degenerated muscle fibers were identified by IgG fluorescein isothiocyanate conjugate antibody (anti-mouse; Sigma-Aldrich), following the protocol of previous study [32]. Normal and degenerated fibers were quantified to projection the total number of fibers in each muscle.

### Regenerated muscle fibers

The regenerated muscle fibers were characterized due to the presence of a central nucleus [33] and the normal fibers showed a peripheral nucleus. Normal and regenerated fibers were quantified to projection the total number of fibers in each muscle.

### Minimum diameter of Feret

Approximately 100 minimum distances of parallel tangents at the opposite edges of the muscle fiber [34], distributed in random fields, were analyzed by Image-Pro Plus 6.0 software (Media Cybernetic, Silver Spring, MD, USA), following the protocol of previous study [29].

### Fibrosis quantification

Cryosection of quadriceps muscles were stained with Masson’s trichrome for quantitative observation of fibrosis, as previously reported [35]. The fibrosis area and the entire cross-section area were quantified by ImagePro-Express software (Media Cybernetic; Silver Spring, MD) and results were expressed as the percentage of fibrosis in relation to the total cross-section area.

### Western blotting

Protein quantification by western blotting was based on the protocol previously described [24, 27, 32]. Briefly, samples of the primary skeletal muscle cells and the quadriceps muscles were homogenized, and the extracts obtained were centrifuged and the supernatant was used for analysis of the total extract method. Later, the protein was applied on SDS-polyacrylamide gel in a Bio-Rad electrophoresis device, and the gel electrotransfer to the nitrocellulose membrane was performed in a Mini Trans-Blot® transfer device. Next, the membranes were incubated with primary antibodies and the next day were exposed to Clarity Western ECL solution, followed by exposure in the G-Box Chamber to identified immunoreactive bands. Gene Tools from the Syngene program was used to quantify the optical densitometry. β-actin antibody was used as an internal control.

Primary antibodies: LC3B (Cell-Sinaling D3U4C); SQSTM1/p62 (Cell-Sinaling D1Q5S); Beclin-1 (Cell-Sinaling D40C5); Parkin (Cell-Sinaling Prk8); AMPK alfa (Invitrogem MA5-14922); Phospo- AMPK alfa Thr172 (Invitrogem PA517831) mTORC1(Cell-Sinaling #2972) NF-kB (BIO-RAD AHP1342); TGFβ-1 (Sigma-Aldrich T0438); MHC*fast* (Sigma-Aldrich M4276); MHC*slow* (Sigma-Aldrich M8421); MyoD (Santa-Cruz m-318); Myogenin (Santa-Cruz F5D). Peroxidase-conjugated secondary antibodies: anti -rabbit (Promega Corporation W4011); anti-mouse (Promega Corporation W4021) and anti-goat (KPL 14-13-06).

### Gene Expression by Quantitative Real-Time PCR

Gene Expression by Quantitative Real-Time PCR was based on the protocol previously described [27]. Briefly, total RNA was extracted using Trizol; RNA quantification was performed by spectrophotometry; and the synthesis of complementary DNA (cDNA) was performed from total RNA using the High-Capacity cDNA Kit. The ABI 7500 One Step quantitative PCR system was used to quantify the gene expression. Primer sequences are listed in Table 2.

**Table 2:** Primer sequences for RT-PCR.

Primer	Sequence 5’-3’
**Ribosomal Protein L39 (RPL39)**	F: 5’ -C AAAATCGCCCTATTCCTCA-3’ R: 5’ -AGACCCAGCTT CGTTCTCCT-3’
**MyoD1**	F: 5’-CTGCTCTGATGGCATGATTGGA-3’ R:5’ –CACTGTAGTAGGCGGTGTCG-3’
**Myogenin**	F: 5’ –GTCCCAACCCAGGAGATCATTT-3’ R: 5’ –CGATGGACGTAAGGGAGTGC-3’

### Statistical analysis

One-way ANOVA followed by Tukey test was used to statistical analysis. Significant differences were closed as p < 0.05. The results obtained are expressed as mean ± standard deviation (SD).

## Results

### *In vitro* studies

#### Muscle cell morphology and diameter

**Fig 1:**
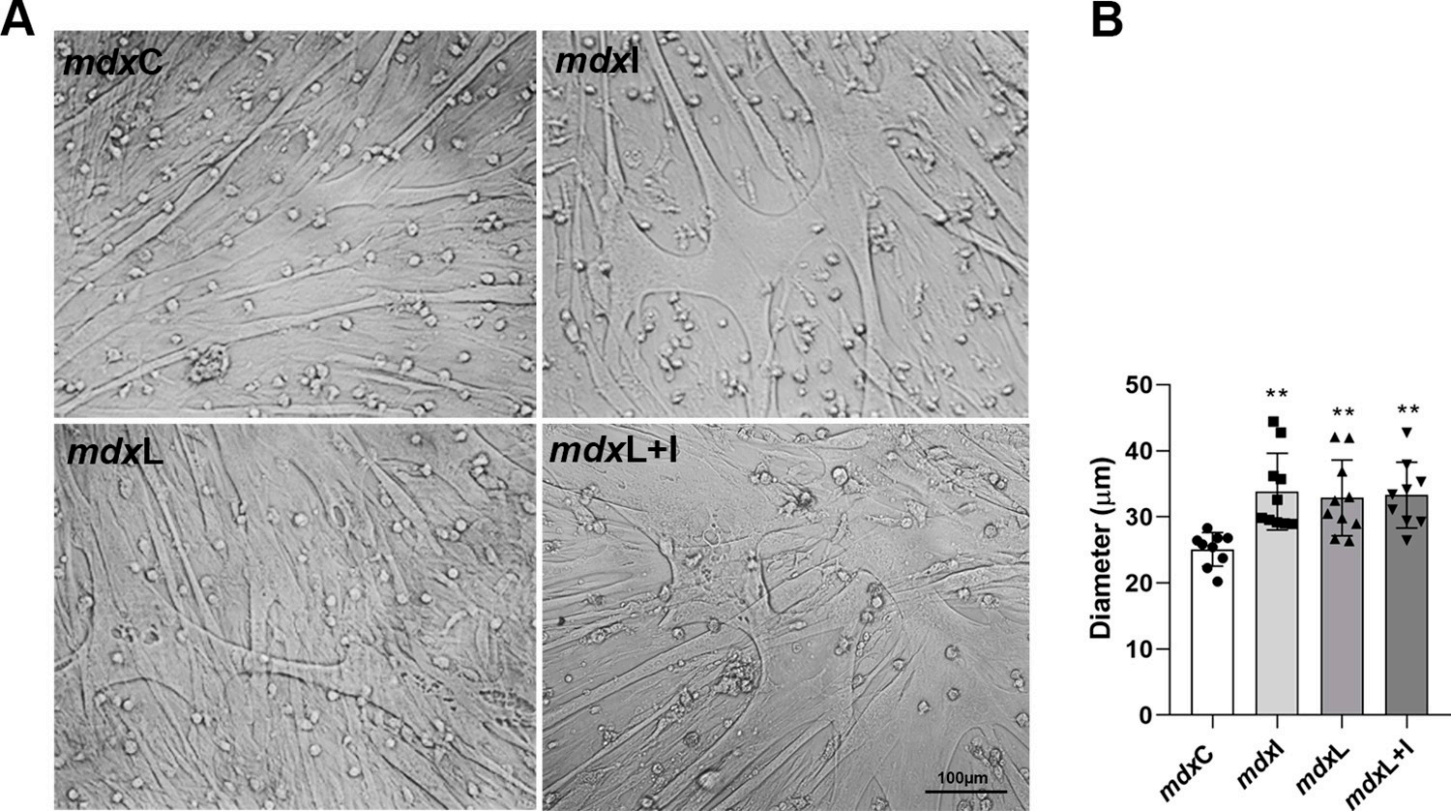
Muscle morphology and diameter: *In vitro* studies. **(A)** Images of untreated *mdx* muscle cells (mdxC), mdx muscle cells treated with Idebenone (mdxI); mdx muscle cells treated with LEDT (mdxL) and mdx muscle cells treated with Idebenone and LEDT (mdxL+I). Scale bar 100 μm, 20x. **(B)** The graphs show the muscle fibers diameter (μm) of all experimental groups. All data are expressed by mean±SD and the experiments were carried out in triplicate. **P< 0.001 versus mdxC. One-way ANOVA followed by Tukey post test was used for statistical analysis.

To detect the potential cytotoxicity of LEDT and Idebenone treatment, applied alone or together, we analyzed the morphological characteristics of muscle cells. The results demonstrated that all treatments were not cytotoxic to the dystrophic muscle cells, since the untreated mdx and treated (IDE; LEDT; LEDT + IDE) muscle cell showed the same morphology, observed by thick and branching myotubes (Fig 1A). The muscle diameter was also evaluated, and a significant increase in diameter of the mdx muscle cells after all treatments was also observed (by mdxI 34.7%; mdxL 31.0%; and mdxL+I 32.7%; Fig 1B). In addition, the dose-dependent toxicities of Idebenone and LEDT on control (C57BL/10) muscle cells, by MTT assay, was showed in S1 Fig.

#### Autophagy-mitophagy markers

To analyze the role of autophagy-mitophagy in dystrophic muscle cells untreated and treated (IDE; LEDT; LEDT+IDE), the panel of four markers, namely LC3 (I/II), Sequestosome 1 (SQSTM1/p62), Beclin-1, and Parkin were evaluated.

**Fig 2:**
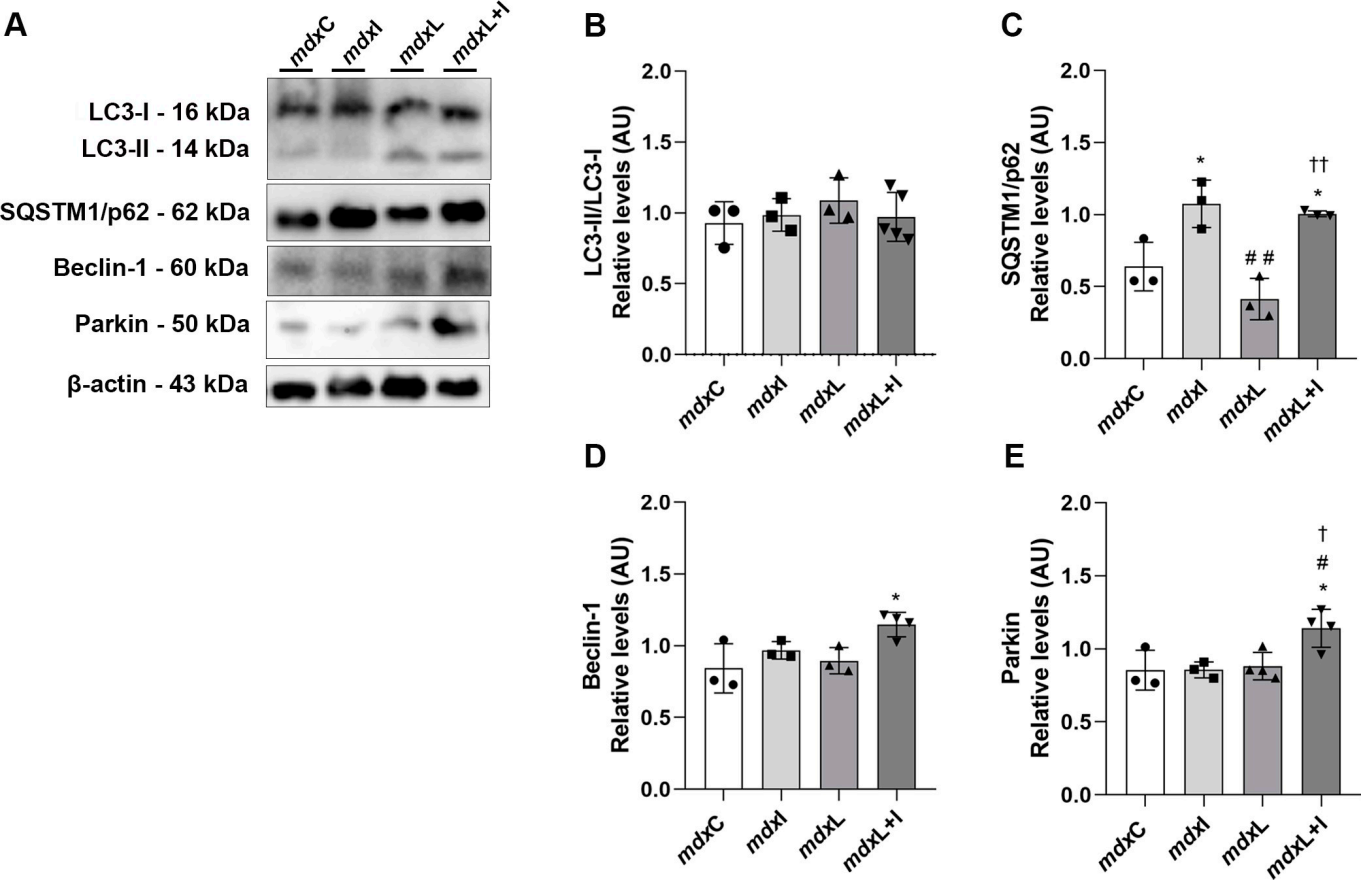
Autophagy-mitophagy markers: *In vitro* studies. **(A)** LC3 (II/I), SQSTM1/p62, Beclin and Parkin western blotting data in untreated *mdx* muscle cells (mdxC), mdx muscle cells treated with Idebenone (mdxI); mdx muscle cells treated with LEDT (mdxL) and mdx muscle cells treated with Idebenone and LEDT (mdxL+I). **(B)** LC3 (II/I); **(C)** SQSTM1/p62; **(D)** Beclin; and **(E)** Parkin graphs from mdxC, mdxI, mdxL and mdxL+I groups. Loading control: β-actin. All data are expressed by mean±SD and the experiments were carried out in triplicate. *P< 0.05 versus mdxC; ^#^P< 0.05 versus mdxI; ^# #^P< 0.01 versus mdxI; ^†^ P< 0.05 versus mdxL; ^††^ P< 0.01 versus mdxL. One-way ANOVA followed by Tukey post test was used for statistical analysis.

LC3 is detected as two bands following immunoblotting: one represents LC3-I, which is cytosolic, and the other LC3-II, which is present in isolation membranes and autophagosomes. The amount of LC3-II is closely related to the number of autophagosomes, serving as a good indicator of autophagosome formation [36]. Western blot analysis showed the LC3 band at 14 kDa (LC3-II) and 16 kDa (LC3-I; Fig 2A). Under our experimental conditions no significant difference in the LC3 (II/I) levels was observed between the experimental groups (Fig 2A and 2B).

We also monitored the accumulation of the sequestosome1 (SQSTM1), also called p62 that links ubiquitinated proteins to the autophagic machinery for degradation [37]. We observed a prominent band of SQSTM1/p62 at 62 kDa (Fig 2A). The SQSTM1/p62 levels were significantly reduced in the mdx muscle cells treated with LEDT (by 35.0%) compared to the untreated mdx muscle cells (Fig 2A and 2C). On the other hand, the mdx muscle cells treated with IDE and/or LEDT+IDE showed a significant increase in SQSTM1/p62 levels (by 69.8% and 58.7%, respectively), compared to the untreated mdx muscle cells (Fig 2A and 2C).

The LEDT and Idebenone, applied together, also led to the up-regulation of proteins involved in the mitophagy pathway. The Beclin-1 and Parkin levels were significantly increased in the mdx muscle cells treated with LEDT+IDE (by 35.7% and 34.1%, respectively) compared to the untreated mdx muscle cells (Fig 2A, 2D and 2E).

Original images underlying all blot and representative membranes stained with Ponceau were showed in S2 Fig.

#### Regulation of autophagy/signaling pathways

In skeletal muscles, autophagy is under the tight regulation of several signaling. Here, the 5’-adenosine monophosphate-activated protein kinase (AMPK); mammalian target of rapamycin complex 1 (mTORC1); nuclear factor κB (NF-κB); and transforming growth factor (TGF)-β signaling pathways were evaluated.

**Fig 3:**
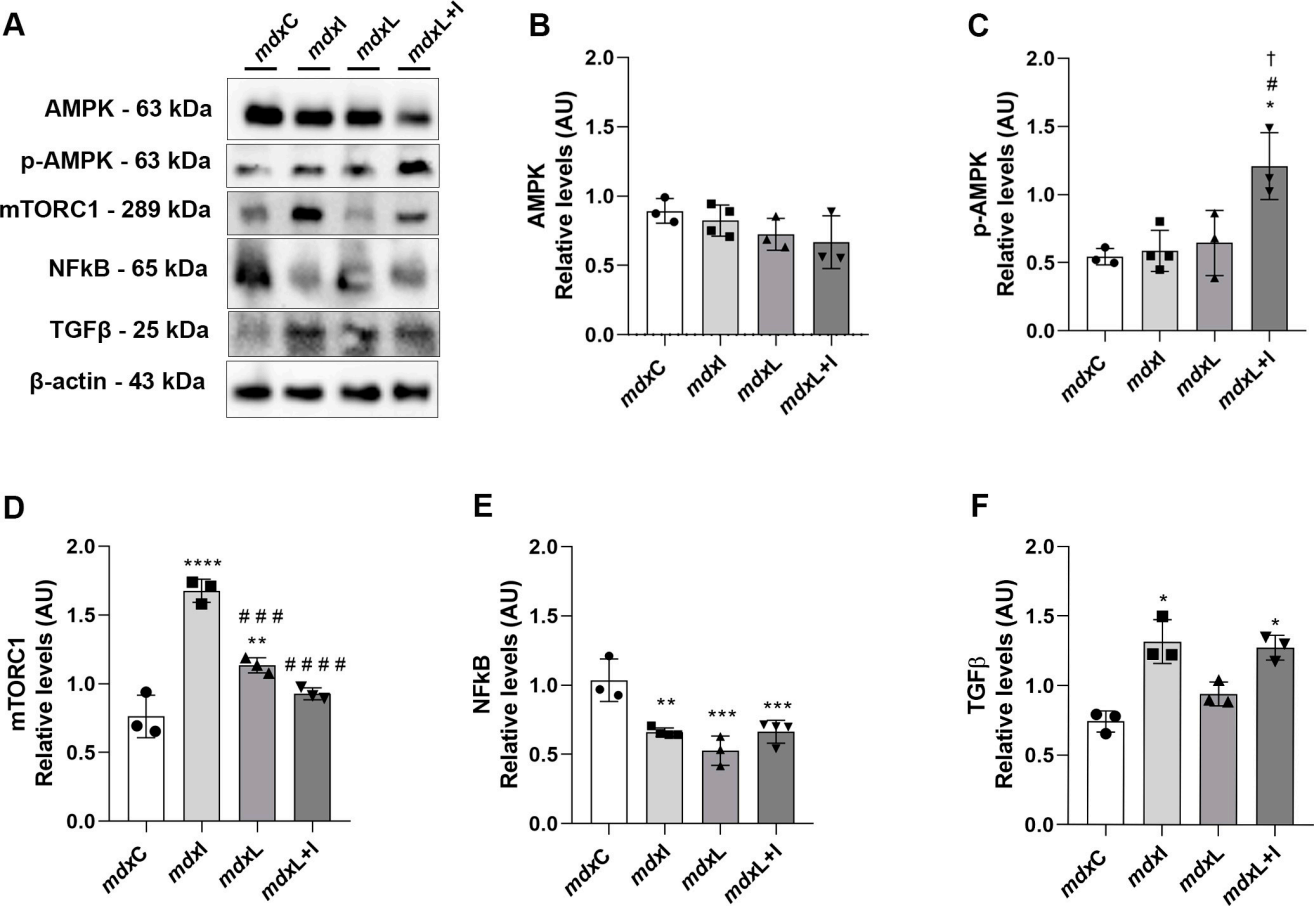
Regulation of autophagy/signaling pathways: *In vitro* studies. **(A)** AMPK, p-AMPK, m-TORC1, NF-κB and TGF-β western blotting data in untreated *mdx* muscle cells (mdxC), mdx muscle cells treated with Idebenone (mdxI); mdx muscle cells treated with LEDT (mdxL) and mdx muscle cells treated with Idebenone and LEDT (mdxL+I). **(B)** AMPK; **(C)** p-AMPK; **(D)** m-TORC1; **(E)** NF- κB; and **(F)** TGF-β graphs from mdxC, mdxI, mdxL and mdxL+I groups. Loading control: β-actin. All data are expressed by mean±SD and the experiments were carried out in triplicate. *P< 0.05 versus mdxC; **P< 0.01 versus mdxC; ***P< 0.001 versus mdxC; ^#^P< 0.05 versus mdxI; ^# # #^P< 0.001 versus mdxI; ^# # #^P< 0.001 versus mdxI; ^†^ P< 0.05 versus mdxL. One-way ANOVA followed by Tukey post test was used for statistical analysis.

No significant difference in AMPK levels was observed between the experimental groups (Fig 3A and 3B). In contrast, the p-AMPK levels were significantly increased in the mdx muscle cells treated with LEDT+IDE (by 124.0%) compared to the untreated mdx muscle cells (Fig 3A and 3C). The mTORC1 levels were significantly increased in the mdx muscle cells treated with IDE and LEDT (by 119.7% and 48.7%, respectively) compared to the untreated mdx muscle cells (Fig 3A and 3D). The NF-κB levels were significantly reduced in the mdx muscle cells treated with IDE, LEDT and LEDT+IDE (by 35.9%, 49.5% and 36.0%, respectively) compared to the untreated mdx muscle cells (Fig 3A and 3E). In relation to TGF-β levels (Fig 3A and 3F), it was observed a significant increase in its levels in the mdx muscle cells treated with IDE and LEDT+IDE (by 77.0% and 71.6%, respectively) compared to the untreated mdx muscle cells (Fig 3A and 3E).

Original images underlying all blot and representative membranes stained with Ponceau were showed in S2 Fig.

#### Muscle cell differentiation regulators

**Fig 4:**
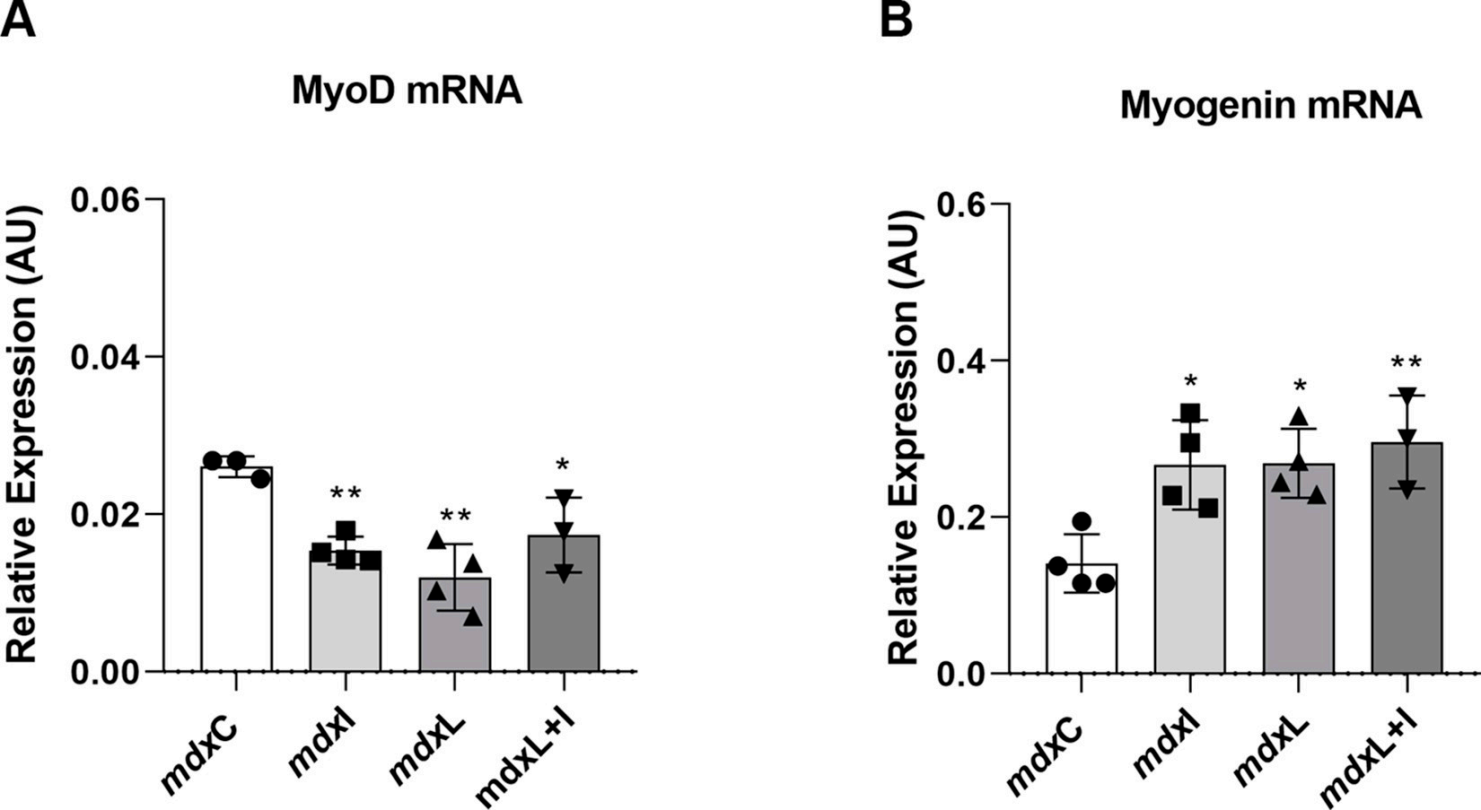
Muscle cell differentiation regulators: *In vitro* studies. Gene expression of MyoD (A) and Myogenin (B) by qRT-PCR in untreated *mdx* muscle cells (mdxC), mdx muscle cells treated with Idebenone (mdxI); mdx muscle cells treated with LEDT (mdxL) and mdx muscle cells treated with Idebenone and LEDT (mdxL+I). All data are expressed by mean±SD and the experiments were carried out in triplicate. *P< 0.05 versus mdxC; **P< 0.01 versus mdxC. One-way ANOVA followed by Tukey post test was used for statistical analysis.

The MyoD mRNA relative expression was significantly lower in the mdx muscle cells treated with IDE (by 42.3%); LEDT (by 53.8%) and LEDT+IDE (by 34.6%) compared to the untreated mdx muscle cells (Fig 4A).

On the other hand, the mRNA relative expression of myogenin was significantly higher in the mdx muscle cells treated with IDE (by 85.7%); LEDT (by 85.7%) and LEDT+IDE (by 107.1%) compared to the untreated mdx muscle cells (Fig 4B).

These findings are very interesting, since MyoD is normally associated with faster and myogenin with slower muscle fiber types [38].

### *In vivo* studies

#### Muscle weight and strength

Total experimental groups gained weight during the experimental period (by 88.5% in the Ctrl group; 42.8% in the mdxS group; 43.1% in the mdxL group, group; 49.2% in the mdxI group; and 62.7% in the mdxL+I group; Table 3). Concomitantly, it was observed a significant weight gain in the quadriceps muscle in mdx mice treated with LEDT (by 193.7%); IDE (by 156.25%); and LEDT+IDE (by 112.6%) compared to the untreated mdx mice (Table 3).

**Table 3:** Morphological and biochemical parameters.

	Ctrl		mdxS		mdxL		mdxI		mdxL+I	
Weight and force parameters										
	*Start*	*End*	*Start*	*End*	*Start*	*End*	*Start*	*End*	*Start*	*End*
Body weight (g)	7.0±0.7	13.2±1.2	5.6±0.3 ***°°°°***	8.0±0.9 ***°°°°***	5.1±0.3 ***°°°°***	7.3±1.2 ***°°°°***	6.3±0.2 °° * ^†††^	9.4±0.°°°° * ^†††^	5.9±0.5 °°°^†^	9.6±0.8 °°°°*^†††^
Body weight/ period (%)	88.5		42.8		43.1		49.2		62.7	
Force/Body weight (g/g)	1.3±0.1	1.6±0.01	1.4±0.2	1.4±0.2	1.4±0.2	1.5±0.1	1.4±0.1	1.4±0.2	1.4±0.1	1.5±0.1
Force gain/period (%)	23.0		0		7.1		0		7.1	
Quadriceps mass (g/g)	0.01059±0.1		0.003226±0.1 ***°°°°***		0.009493±0.1 ****		0.008242±0.09 ****		0.006806±0.2 °°***^#^	
**CK parameters**										
Creatine Kinase levels—plasma (U/L)	325.0±173.9		3613±477.9 ***°°°°***		3368±1079 ***°°°°***		972.7±465.5 ****^††††^		3105±624.2 ***°°°°***^# # # #^	
Creatine Kinase levels—muscle (U/L)	2665± 751.2		983.1± 92.08 °°		1079±206.9 °°°		1514±213.3 °		4266±414.6 °°°****^††††^^# # # #^	

Body weight (g) was quantified at the beginning and after 14 days of LED and IDE treatment. Forelimb muscle strength was evaluated by taking measurements of force at time points Start and End, and normalized by body weight. Quadriceps mass was normalized to the final body weight. Creatine kinase levels were obtained at the end of the experiment. Experimental groups: C57BL/10 mice (Ctrl); dystrophic mice received sham LEDT and carboxymethylcellulose sodium salt diluted in water (mdxS); dystrophic mice treated with LEDT (mdxL); dystrophic mice treated with IDE (mdxI); and dystrophic mice treated with LEDT and IDE (mdxL+I). All values are shown as the mean ± standard deviation (SD).

° P≤ 0.05 versus Crtl

°° P≤ 0.001 versus Crtl

°°° P≤ 0.0001 versus Crtl

*°°°°* P ≤ 0.0001 versus Crtl

* P ≤ 0.05 versus mdxS

*** P ≤ 0.0001 versus mdxS

**** P ≤ 0.00001 versus mdxS

^†^ P ≤ 0.05 versus mdxL

^†††^ P≤ 0.0001 versus mdxL

^††††^ P≤ 0.00001 versus mdxL

^#^ P≤ 0.05 versus mdxI

^# # # #^ P≤ 0.05 versus mdxI. One-way ANOVA followed by Tukey post test was used for statistical analysis.

The Ctrl; mdxL; and mdxL+I groups showed an increase in force gain (by 23%, 7.1% and 7.1%, respectively) in the experimental period (Table 3).

#### Degeneration/regeneration process

A significant reduction in CK (serum) levels (by 73.0%) was identified in blood samples of the mdx mice treated with IDE (Table 1) compared to the untreated mdx mice (Table 3). In relation to CK in the quadriceps muscle tissue specimens, a significant increase in its levels (by 334.0%) was observed in mdx mice treated with LEDT+IDE compared to the untreated mdx mice (Table 3).

**Fig 5:**
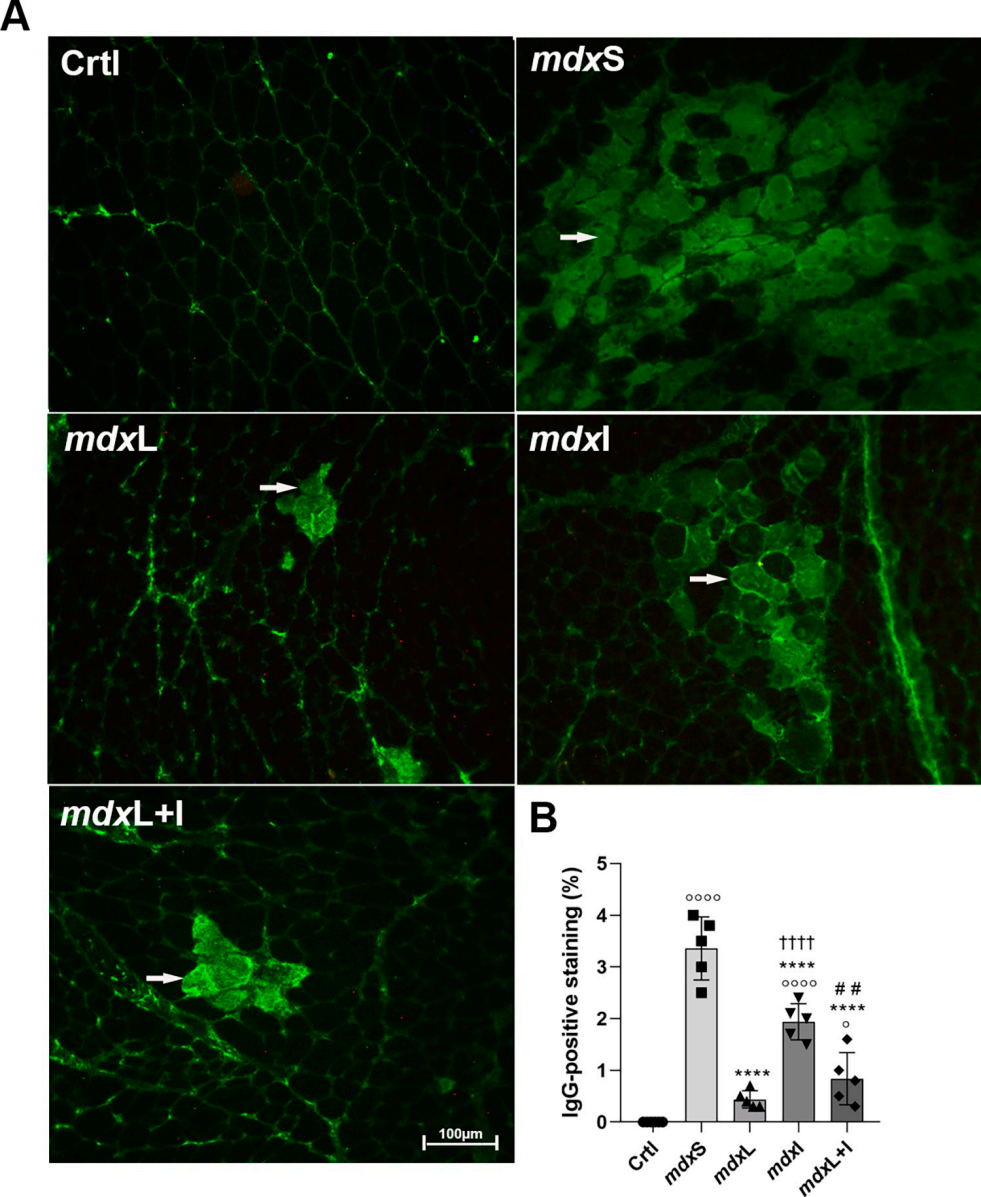
Muscular degeneration process: *In vivo* studies. **(A)** Cross sections of quadriceps muscle showing IgG staining (white arrow) in normal untreated C57BL/10 mice (Ctrl); dystrophic mice received sham LEDT and carboxymethylcellulose sodium salt diluted in water (mdxS); dystrophic mice treated with LEDT (mdxL); dystrophic mice treated with Idebenone (mdxI); and dystrophic mice treated with LEDT and Idebenone (mdxL+I). Scale bar 100 μm, 20x. **(B)** The graph shows the IgG staining (%) in all experimental groups. All data are expressed by mean±SD.° P< 0.05 versus Ctrl°°°° P< 0.00001 versus Ctrl; ****P< 0.00001 versus mdxC; ^††††^ P< 0.00001 versus mdxL; ^# #^P< 0.01 versus mdxI. One-way ANOVA followed by Tukey post test was used for statistical analysis.

Sarcolemmal integrity leakage was identified in quadriceps muscles of experimental groups by intracellular fiber staining for IgG antibody (Fig 5A). The mdxL, mdxI and mdxL+I groups showed a significant reduction in the number of degenerated muscle fibers (by 87.0%, 42.3% and 75.0%; respectively) compared to mdxS group (Fig 5B). Representative quadriceps cross-sections showing intracellular fiber staining for IgG antibody in the 5 animals from each experimental group were showed in S3 Fig.

**Fig 6:**
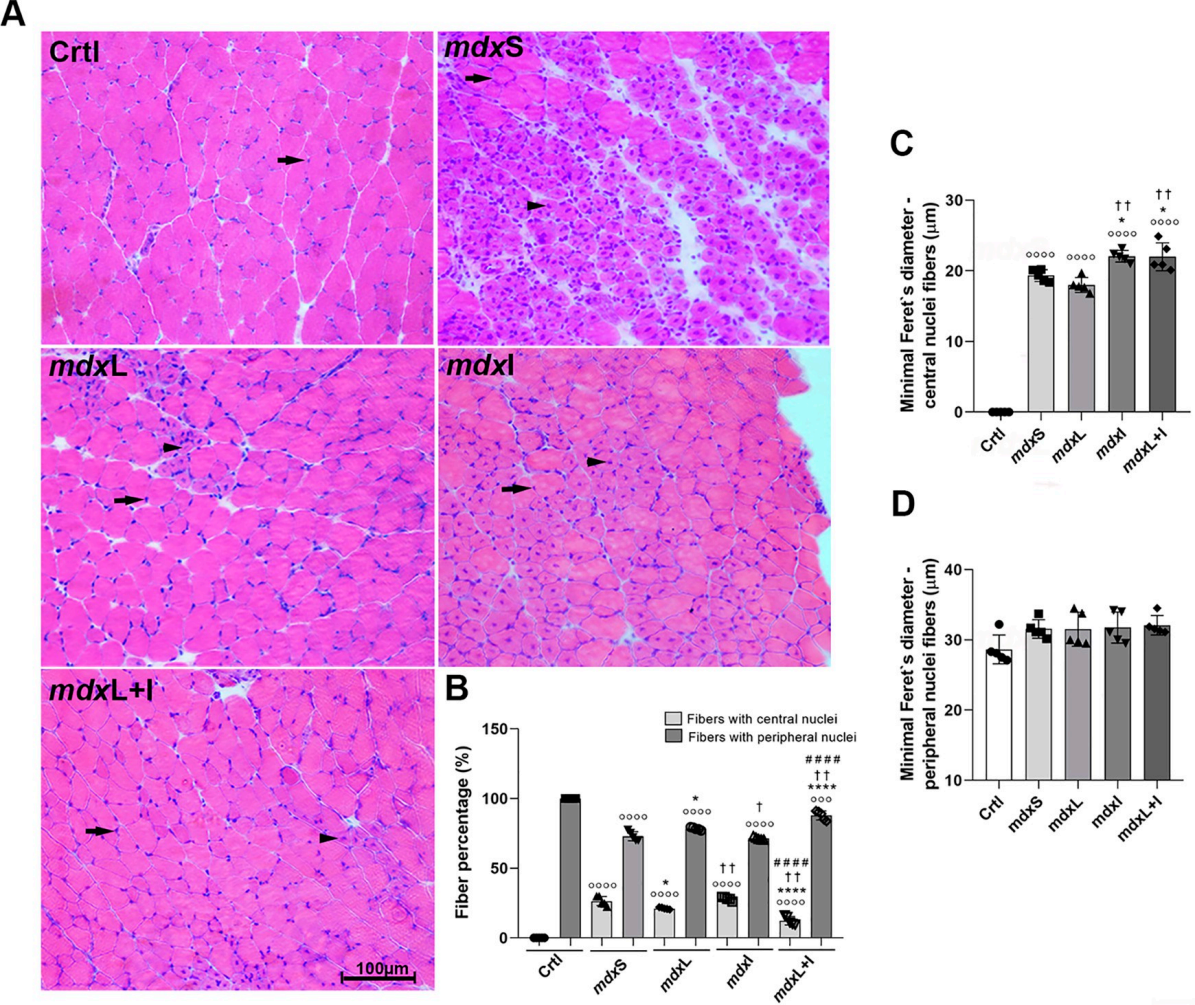
Muscular regeneration process: *In vivo* studies. (**A**) Cross sections of quadriceps muscle showing fibers with central nuclei (black arrowheads) and with peripheral nuclei (black heads) in normal untreated C57BL/10 mice (Ctrl); dystrophic mice received sham LEDT and carboxymethylcellulose sodium salt diluted in water (mdxS); dystrophic mice treated with LEDT (mdxL); dystrophic mice treated with Idebenone (mdxI); and dystrophic mice treated with LEDT and Idebenone (mdxL+I). Scale bar 100 μm, 20x. (**B**) The graph shows the percentage of fibers with central nuclei fibers and fibers with peripheral nuclei. (**C**) The graph shows the minimal Feret’sdiameter (μm) of fibers with central nuclei. (**D**) The graph shows the minimal Feret’sdiameter (μm) of fibers with peripheral nuclei. All data are expressed by mean±SD.°°° P< 0.001 versus Ctrl°°°° P< 0.00001 versus Ctrl; *P< 0.05 versus mdxC; ****P< 0.00001 versus mdxC; ^†^ P< 0.05 versus mdxL; ^††^ P< 0.01 versus mdxL; ^# # # #^P< 0.00001 versus mdxI. One-way ANOVA followed by Tukey post test was used for statistical analysis.

Regenerated muscle fibers and normal muscle fibers were found in the dystrophic quadriceps muscle (Fig 6A). The mdxL and mdxL+I groups showed a significant reduction in the number of regenerated muscle fibers (by 21.6% and 52.2%; respectively) compared to mdxS group (Fig 6B). Concomitantly, the mdxL and mdxL+I groups showed an increase in the number of normal muscle fibers (by 8.0% and 19.8%; respectively) compared to mdxS group (Fig 6B). A significant increase in regenerated muscle fibers diameter was observed in mdx mice treated with Idebenone (by 17.9%) and LEDT+Idebenone (by 12.0%), compared to the untreated mdx mice (Fig 6C). No significant difference in normal muscle fibers diameter was observed between the experimental groups (Fig 6D). Representative quadriceps cross-sections showing normal muscle fibers and regenerated muscle fibers in the 5 animals from each experimental group were showed in S4 Fig.

**Fig 7:**
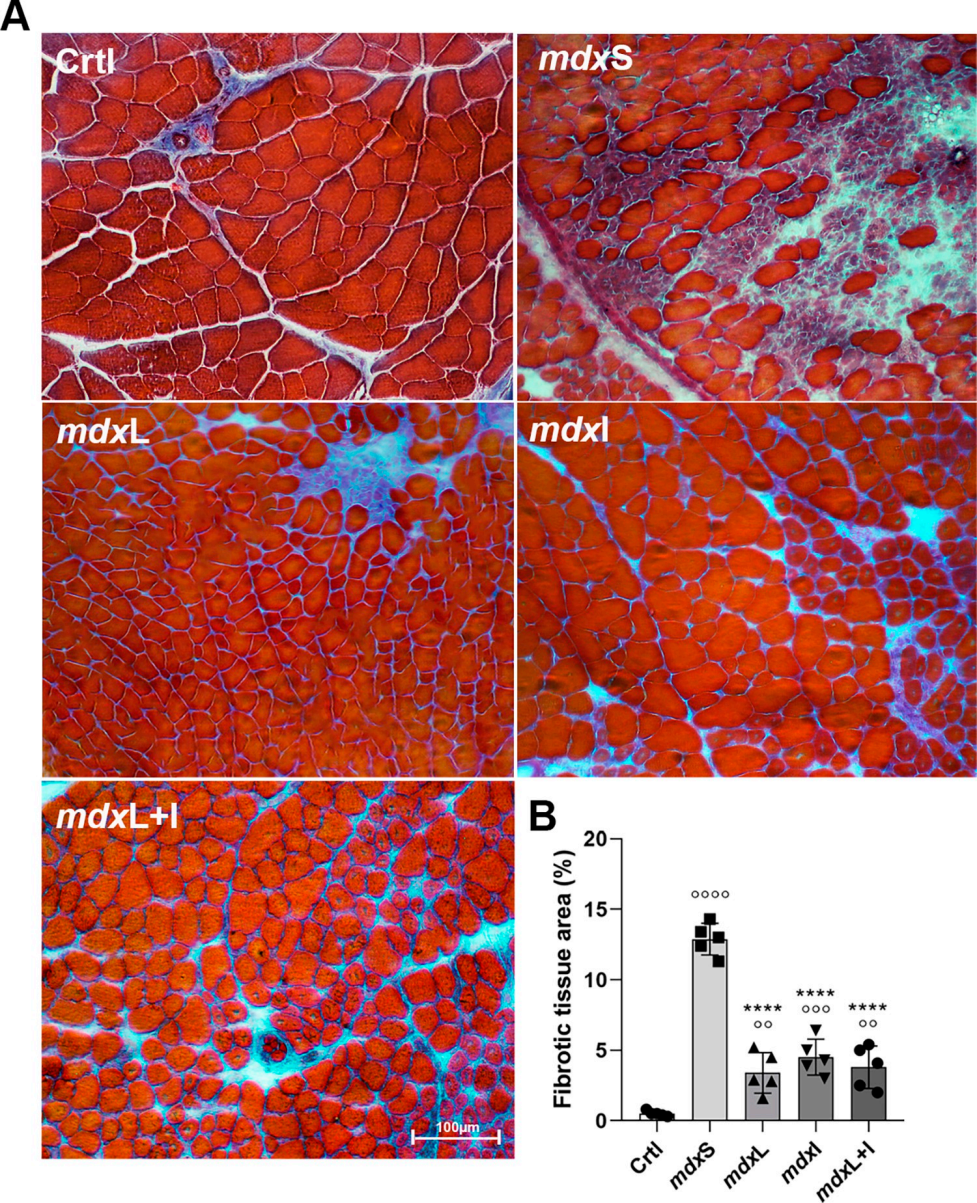
Fibrotic area: *In vivo* studies. (**A**) Cross sections of quadriceps muscle showing fibrosis area (blue color) in normal untreated C57BL/10 mice (Ctrl); dystrophic mice received sham LEDT and carboxymethylcellulose sodium salt diluted in water (mdxS); dystrophic mice treated with LEDT (mdxL); dystrophic mice treated with Idebenone (mdxI); and dystrophic mice treated with LEDT and Idebenone (mdxL+I). Scale bar 100 μm, 20x. (**B**) The graph shows the percentage of fibrosis area. All data are expressed by mean±SD.°° P< 0.01 versus Ctrl°°° P< 0.001 versus Ctrl°°°° P< 0.00001 versus Ctrl; ****P< 0.00001 versus mdxC. One-way ANOVA followed by Tukey post test was used for statistical analysis.

The fibrotic area was identified in the quadriceps muscles of the experimental groups by Masson trichrome staining (Fig 7A). The fibrotic area was significantly increased in the quadriceps muscle of the mdxS group (by 2476.0%) compared to the control group (Fig 7B). The mdxL, mdxI and mdxL+I groups showed a significant reduction in the fibrotic area in the quadriceps muscles (by 73.6%; 64.9%; and 70.49%; respectively) compared to the control and mdxS groups (Fig 7B). Representative quadriceps cross-sections showing fibrotic area in the 5 animals from each experimental group were showed in S5 Fig.

### Muscle cell differentiation regulators

**Fig 8:**
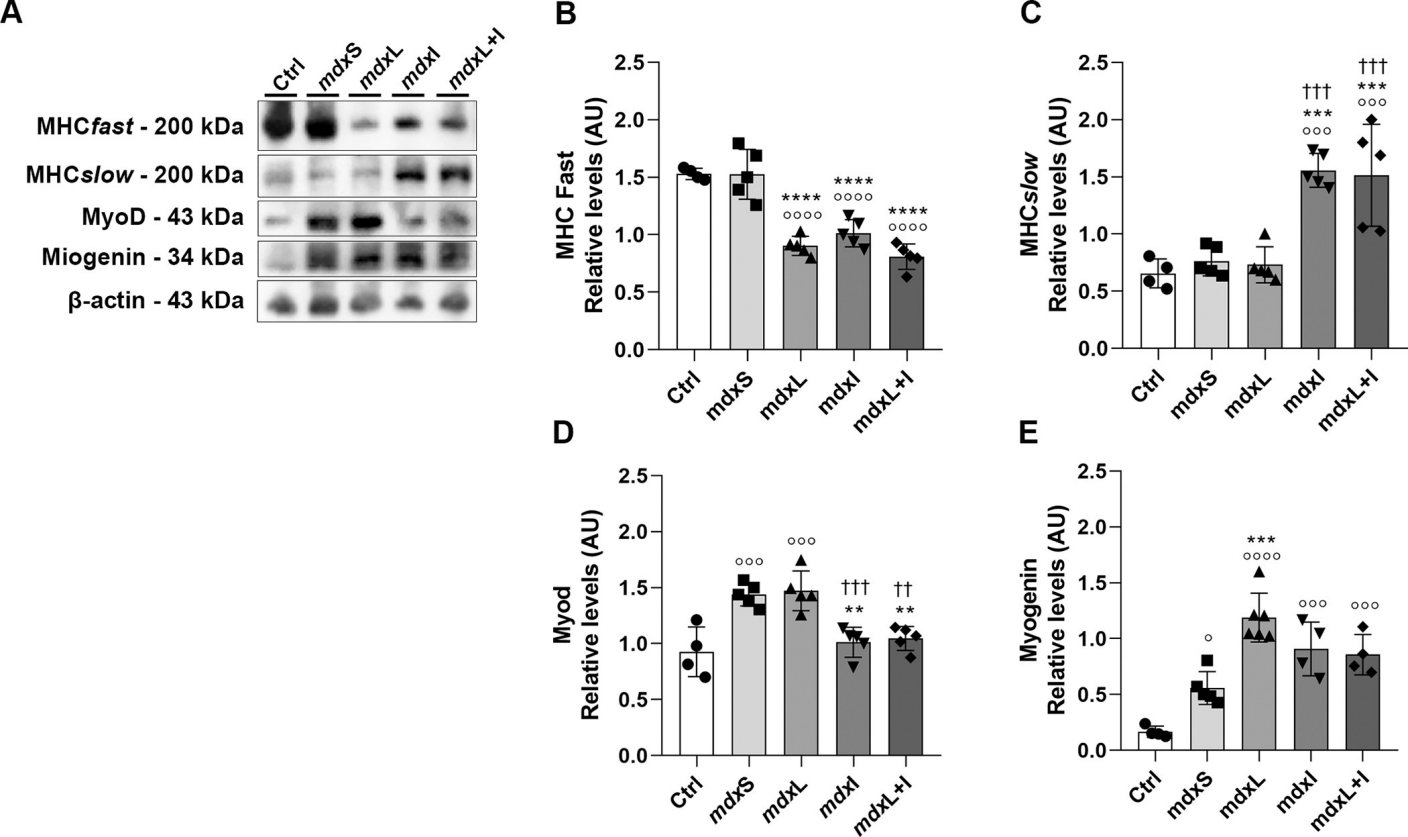
Muscle cell differentiation regulators: *In vivo* studies. **(A)** MHC-fast, MHC-slow, Myo-D, and myogenin western blotting data in normal untreated C57BL/10 mice (Ctrl); dystrophic mice received sham LEDT and carboxymethylcellulose sodium salt diluted in water (mdxS); dystrophic mice treated with LEDT (mdxL); dystrophic mice treated with Idebenone (mdxI); and dystrophic mice treated with LEDT and Idebenone (mdxL+I). **(B)** MHC-fast; **(C)** MHC-slow; **(D)** Myo-D; and **(E)** myogenin graphs from Ctrl, mdxS, mdxL, mdxI and mdxL+I groups. Loading control: β-actin. All data are expressed by mean±SD and the experiments were carried out in triplicate.° P< 0.05 versus Ctrl;°°° P< 0.001 versus Ctrl;°°°° P< 0.00001 versus Ctrl; ***P< 0.001 versus mdxC; ****P< 0.00001 versus mdxC; ^††^ P< 0.01 versus mdxL; ^†††^ P< 0.001 versus mdxL. One-way ANOVA followed by Tukey post test was used for statistical analysis.

Regarding the muscle cell differentiation regulators, the mdxL; mdxI; and mdxL+I groups showed a significant reduction in the MHC-Fast levels in quadriceps muscle (by 43.2%; 38.3%; and 50.6%; respectively) compared to mdxS group (Fig 8A and 8B). On the other hand, the MHC-Slow levels were significantly higher in the mdx muscle cells treated with idebenone (by 103.9%) and LEDT+IDE (by 98.7%) compared to the mdxS group (Fig 8A and 8C).

In addition, the mdxI and mdxL+I groups showed a significant reduction in the Myo-D levels in quadriceps muscle (by 36.8%; and 26.5%; respectively) compared to the mdxS group (Fig 8A and 8D). The myogenin levels were significantly higher in the mdx muscle cells treated with LEDT (by 107.0%) compared to the mdxS group (Fig 8A and 8E).

Original images underlying all blot and representative membranes stained with Ponceau were showed in S6 Fig.

### Autophagy-mitophagy markers

**Fig 9:**
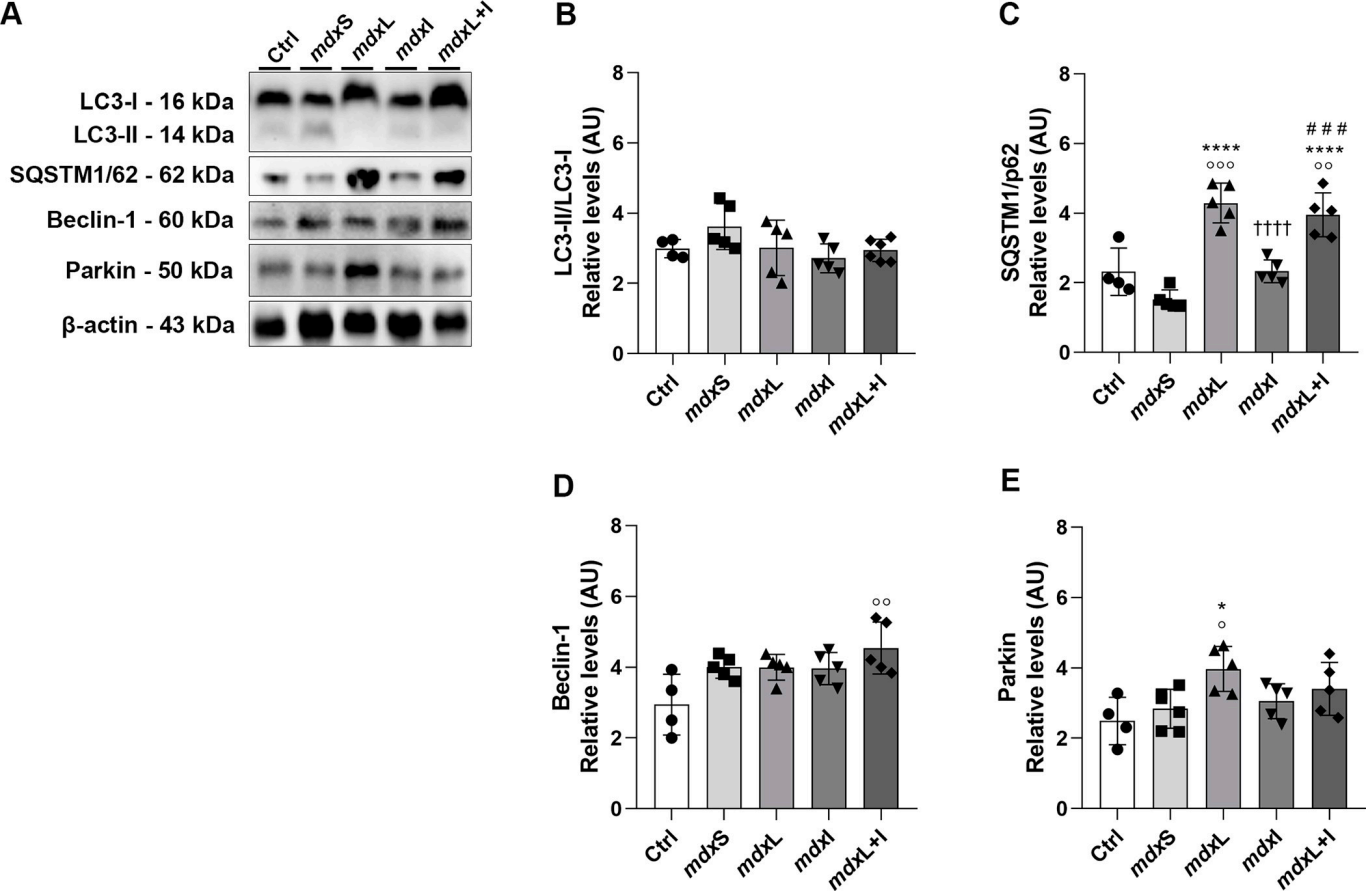
Autophagy-mitophagy markers: *In vivo* studies. **(A)** LC3 (II/I), SQSTM1/p62, Beclin and Parkin western blotting data in normal untreated C57BL/10 mice (Ctrl); dystrophic mice received sham LEDT and carboxymethylcellulose sodium salt diluted in water (mdxS); dystrophic mice treated with LEDT (mdxL); dystrophic mice treated with Idebenone (mdxI); and dystrophic mice treated with LEDT and Idebenone (mdxL+I). **(B)** LC3 (II/I); **(C)** SQSTM1/p62; **(D)** Beclin; and **(E)** Parkin graphs from Ctrl, mdxS, mdxL, mdxI and mdxL+I groups. Loading control: β-actin. All data are expressed by mean±SD and the experiments were carried out in triplicate.° P< 0.05 versus Ctrl;°° P< 0.01 versus Ctrl;°°° P< 0.001 versus Ctrl; **P< 0.01 versus mdxC; *P< 0.05 versus mdxC; ****P< 0.00001 versus mdxC; ^††††^ P< 0.00001 versus mdxL; ^# # # #^P< 0.05 versus mdxI. One-way ANOVA followed by Tukey post test was used for statistical analysis.

No significant difference in the LC3 (II/I) levels was observed between the experimental groups (Fig 9A and 9B). The SQSTM1/p62 levels were significantly increased in the mdx mice treated with LEDT (by 234.3%) and LEDT+IDE (by 182.8%), compared to the mdxS group (Fig 9A and 9C).

Regarding mitophagy markers, Parkin levels were significantly increased in the mdx muscle cells treated with LEDT (40.3%) compared to the control and mdxS groups (Fig 9A and 9E). Already, Beclin levels significantly increased in the mdx muscle cells treated with LEDT+IDE (by 54.4%) compared to the control group (Fig 9A and 9D).

Original images underlying all blot and representative membranes stained with Ponceau were showed in S7 Fig.

### Regulation of autophagy/signaling pathways

**Fig 10:**
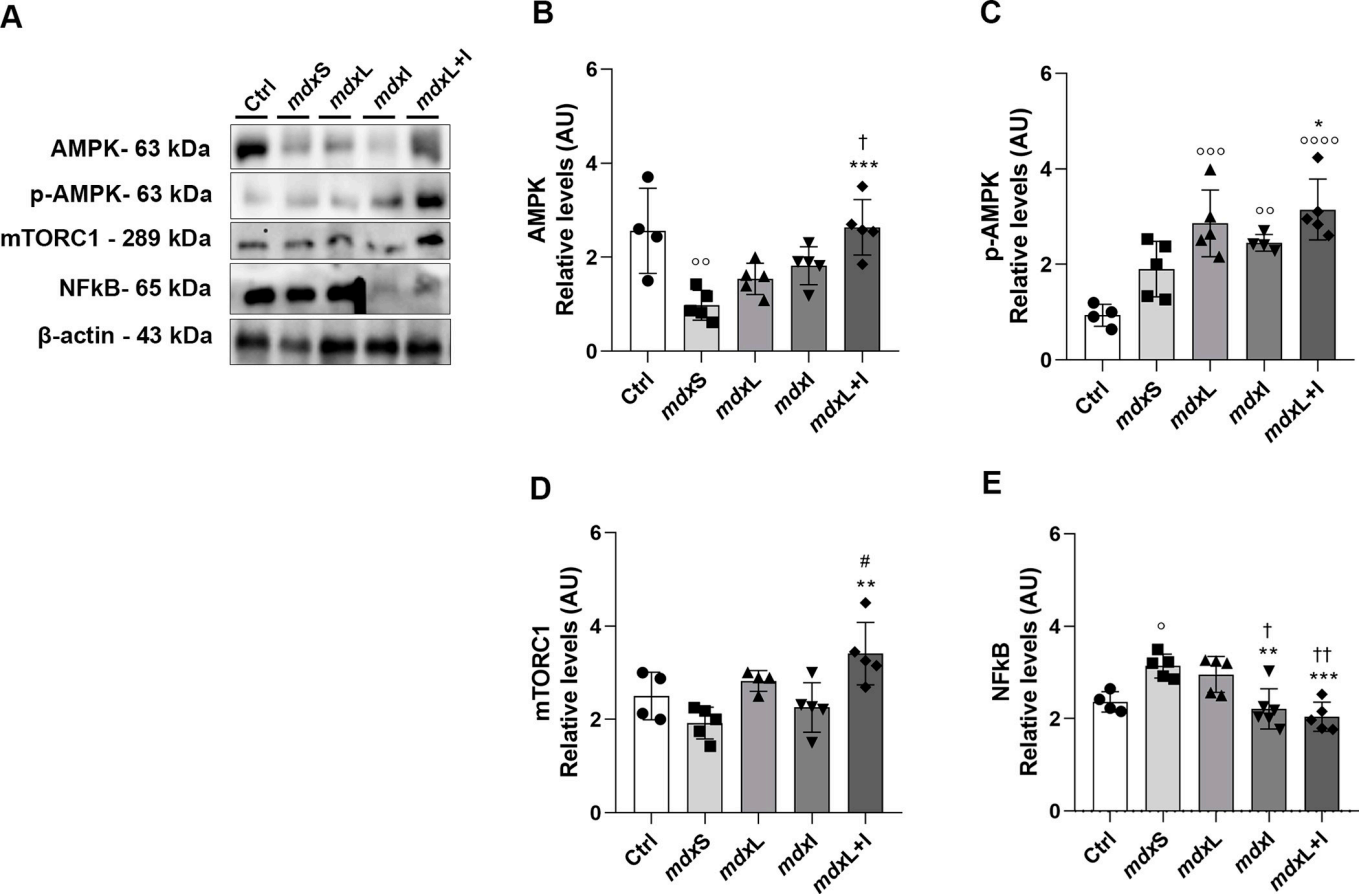
Regulation of autophagy/signaling pathways: *In vivo* studies. **(A)** AMPK, p-AMPK, m-TORC1 and NF-κB western blotting data in normal untreated C57BL/10 mice (Ctrl); dystrophic mice received sham LEDT and carboxymethylcellulose sodium salt diluted in water (mdxS); dystrophic mice treated with LEDT (mdxL); dystrophic mice treated with Idebenone (mdxI); and dystrophic mice treated with LEDT and Idebenone (mdxL+I). **(B)** AMPK; **(C)** p-AMPK; **(D)** m-TORC1; and **(E)** NF-κB graphs from Ctrl, mdxS, mdxL, mdxI and mdxL+I groups. Loading control: β-actin. All data are expressed by mean±SD and the experiments were carried out in triplicate.° P< 0.05 versus Ctrl;°° P< 0.01 versus Ctrl;°°° P< 0.001 versus Ctrl;°°°° P< 0.00001 versus Ctrl; *P< 0.05 versus mdxC; **P< 0.01 versus mdxC; ***P< 0.001 versus mdxC; ^†^ P< 0.05 versus mdxL; ^††^ P< 0.01 versus mdxL; ^#^P< 0.05 versus mdxI. One-way ANOVA followed by Tukey post test was used for statistical analysis.

AMPK and p-AMPK levels significantly increased in the mdx muscle cells treated with LEDT+IDE (by 168.4% and 65.3%, respectively) compared to the mdxS group (Fig 10A–10C). Similar results were obtained regarding mTORC1 levels. A significant increase in mTORC1 levels was observed in the mdx mice treated with LEDT+IDE (by 80.0%), compared to the mdxS group (Fig 10A and 10D). The NF-κB levels were significantly reduced in the mdx muscle cells treated with IDE and LEDT+IDE (by 34.0%, and 36.2%, respectively) compared to the mdxS group (Fig 8A and 8E). Original images underlying all blot and representative membranes stained with Ponceau were showed in S8 Fig.

## Discussion

**Fig 11:**
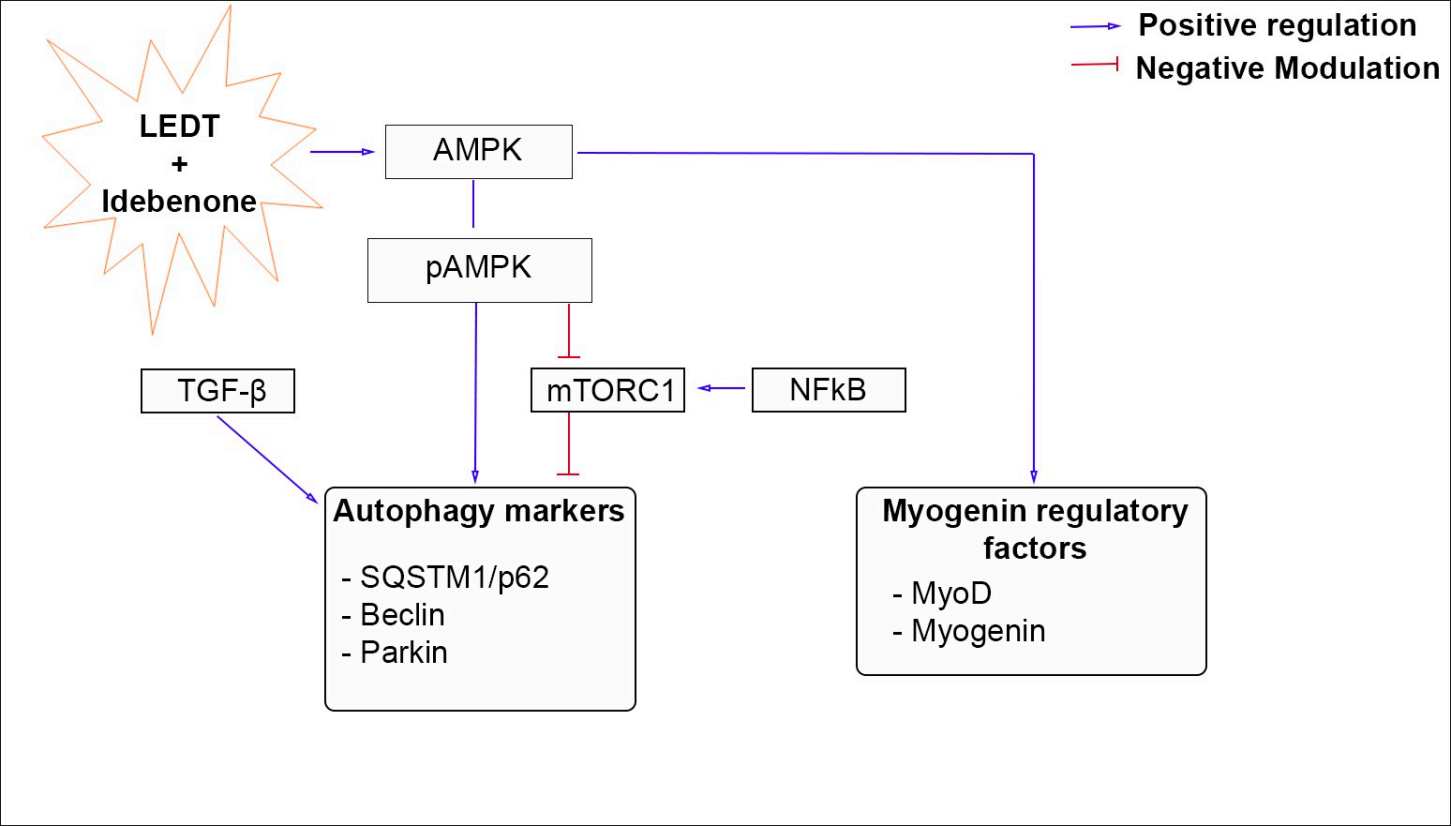
LEDT and Idebenone treatment effects on dystrophic muscle. Blue line: Positive regulation; Red line: Negative modulation.

Several studies have suggested that autophagy plays an essential role in muscle homeostasis maintenance, and its dysregulation has been associated with muscle atrophy and myopathy. In skeletal muscles, autophagy is regulated by strong several signaling inputs and interacts with other signaling pathways [39]. In this study, we detected that LEDT and IDE treatment modulate the autophagic flux by increasing SQSTM1/p62, Beclin and Parkin levels. LEDT and IDE treatment trigger autophagy in dystrophic muscle fibers by up-regulating the AMPK and TGF-β pathways or by suppressing the NF-κB signaling, as summarized in Fig 11.

AMPK signaling pathways play an important role in cell survival, proliferation and metabolism. In addition, the AMPK has some links with the core genes of the autophagyc pathway [40]. We found that LEDT and IDE treatment activated the AMPK pathway, up-regulating p-AMPK levels. In agreement with our results, a previous study showed that PBM triggered the AMPK/PGC-1α pathway to repair mitochondrial bioenergetics and promoted neuroprotective effects after spinal cord injury [40]. It was also reported that AMPK activation encourages autophagy and mitigates muscular dystrophy in the diaphragm muscle from mdx mice [9]. The activation of AMPK induces autophagy through at least two mechanisms in the skeletal muscle: downregulating the mTOR and activating the ULK1 (Unc-51-Like Kinase 1, a mammalian ortholog of Atg1) by direct phosphorylation [13, 41]. In the present study, LEDT and IDE treatment induced autophagy in dystrophic muscle cell fibers, but this was not associated with evidence of mTORC1 inhibition. Thus, we speculate that LEDT+IDE induced autophagy in our experimental conditions may have occurred, at least in part, through an mTOR-independent mechanism. It is possible that this could potentially involve the direct phosphorylation of ULK1 by AMPK, as suggested by previous work [42–45]. Several studies have also demonstrated that autophagy is subjected to the influence of multiple transcription factors, such as TGF-β and NF-κB [39]. The TGF-β has an important role in the regulation of muscle mass, proliferation and differentiation of myoblasts, it has also been reported to activate autophagy [42, 45, 46]. Regarding NF-κB, this factor can both stimulate and inhibit the autophagy (e.g. NF-κB signaling may activate mTOR, promoting the expression of autophagy inhibitor) [39]. In addition, the NF-κB is also related to muscle failure in both physiological and pathophysiological conditions. First, the NF-κB intensifies the expression of proteins related in the ubiquitin-proteasome system, which encourages the loss of skeletal muscle; second, the NF-κB enhances the expression of several proinflammatory cytokine, which aggravate skeletal muscle loss; and finally, the activated NF-κB can hinder the regeneration of myofibers in response to damage [47]. Therefore, our results regarding TGF-β and NF-κB may also contribute to the modulation of autophagy in dystrophic muscle cells treated with LEDT and IDE. In addition, it is important to highlight that the up-regulation of TGF-β [48] and the down-regulation of NF-κB by PBM [28] and IDE [22] have already been observed. However, the understanding of the role of TGF-β and NF-κB in muscle autophagy is still limited, since most studies focus on cancer, and further in-depth investigations are needed.

In the present study, we also found that LEDT and Idebenone treatment improved muscle function and attenuated muscular degeneration in the dystrophic muscle. The treatments lead to an advanced differentiation in the dystrophic muscle, by the down-regulation of MyoD levels and the up-regulation of myogenin levels. These data are in agreement with previous studies, which demonstrated improved myogenin expression in the muscle of the envenomation murine model due to the Bothrops jararacussu snake venom after PBMT [49] and modulation of the myogenic regulatory factors after multiple LEDT wavelengths [28]. In addition, we also identified the down-regulation of MHC-fast levels and up-regulation of MHC-slow levels after LEDT and IDE treatment. This is an interesting finding, since the fast-twitch fibers suffer more damage than the slow-twitch fibers in the dystrophic muscle [50].

These myogenic responses occurred parallel with indicators of augmented autophagy, such as the upregulation of AMPK phosphorylation, as well as elevations in the autophagy marker levels, including LC3, SQSTM1, Beclin, and Parkin. Thus, we speculate that most of the effects of LEDT and IDE treatment on muscle regeneration capacity are mediated by the AMPK pathway. A recent study reported that the AMPK activation in the skeletal muscle of mdx mice evokes several signaling pathways, such as the increase of the autophagy signaling and modulation of the expression of myogenic regulatory factors that promote a disease-resilient phenotype in DMD [51]. In addition, it has been demonstrated that AMPK stimulation led to some beneficial effects in the skeletal muscle of DMD experimental models, including the reduction of skeletal muscle fragility and induction of slow-twitch myogenesis, which make the muscle phenotype more oxidative [52, 53].

Despite the potential effects of treatment with LEDT and Idebenone on dystrophic muscle, the present study has some limitations. First, future studies with wild-type mice subjected to the same therapy with LED irradiation and Idebenone treatment will be useful to understand the beneficial effects of this therapeutic approach on muscle recovery, supplying additional validation for the novel findings of the present work. Second, we analyzed a single application of LEDT and Idebenone treatment in dystrophic muscle cells, which makes it difficult to compare the *in vitro* with the *in vivo* results. Finally, unfortunately, due to technical and logistical factors, it was impossible to carry out Pax7 staining to evaluate if the treatment could also restore the satellite cells pool. However, we intend to investigate this further in future experiments.

To summarize, our study suggests that LEDT and IDE treatments enhance autophagy and prevent muscle degeneration in the dystrophic muscle of the experimental model. The basic mechanism might mainly rely on the activation of AMPK and the associated signaling pathways. These findings, combined with the results of recent studies, reinforce the potential efficacy of LEDT and IDE treatment as an alternative therapy focused on muscle recovery in the dystrophic patient.

## Supporting information

S1 Fig Dose-dependent toxicities of Idebenone and LEDT on control (C57BL/10) muscle cells. MTT assay in untreated control muscle cells (C57BL/10); control muscle cells treated with carboxymethylcellulose sodium salt (Vehicle); control muscle cells treated with different doses of Idebenone; control muscle cells treated with LEDT (LEDT); and control muscle cells treated with LEDT and different doses of Idebenone, after 48h. All data are expressed by mean±SD and the experiments were carried out in triplicate. *P< 0.05 versus C57BL/10; **P< 0.01 versus C57BL/10; ***P< 0.001 versus C57BL/10; ****P< 0.00001 versus C57BL/10;°P< 0.05 versus Vehicle;°°°° P< 0.00001 versus Vehicle; ^φ^P< 0.05 versus Ide 0.03μM. One-way ANOVA followed by Tukey post test was used for statistical analysis.
(TIF)

S2 Fig Original images: Autophagy-mitophagy markers and autophagy/signaling pathways in muscle cells. (A) Representative membranes stained with Ponceau of the different bands. (B) Representative bands for the LC3 (II/I), SQSTM1/p62, Beclin, Parkin, AMPK, p-AMPK, m-TORC1, NF-κB and TGF-β in untreated *mdx* muscle cells (mdxC), mdx muscle cells treated with Idebenone (mdxI); mdx muscle cells treated with LEDT (mdxL) and mdx muscle cells treated with Idebenone and LEDT (mdxL+I). The images in Fig 2B represent 4 independent muscle cell culture per group, in sequence.
(TIF)

S3 Fig Degenerated muscle fibers in quadriceps muscle. Representative quadriceps cross-sections showing intracellular fiber staining for IgG antibody in the 5 animals from each experimental group: C57BL/10 mice (Ctrl); dystrophic mice received sham LEDT and carboxymethylcellulose sodium salt diluted in water (mdxS); dystrophic mice treated with LEDT (mdxL); dystrophic mice treated with Idebenone (mdxI); and dystrophic mice treated with LEDT and Idebenone (mdxL+I). Scale bar 100 μm, 20x.
(TIF)

S4 Fig Regenerated muscle fibers in quadriceps muscle. Representative quadriceps cross-sections showing fibers with central nuclei (black arrowheads) and with peripheral nuclei (black heads) in the 5 animals from each experimental group: C57BL/10 mice (Ctrl); dystrophic mice received sham LEDT and carboxymethylcellulose sodium salt diluted in water (mdxS); dystrophic mice treated with LEDT (mdxL); dystrophic mice treated with Idebenone (mdxI); and dystrophic mice treated with LEDT and Idebenone (mdxL+I). Scale bar 100 μm, 20x.
(TIF)

S5 Fig Fibrotic area in quadriceps muscle. Representative quadriceps cross-sections showing fibrosis area (blue color) in the 5 animals from each experimental group: C57BL/10 mice (Ctrl); dystrophic mice received sham LEDT and carboxymethylcellulose sodium salt diluted in water (mdxS); dystrophic mice treated with LEDT (mdxL); dystrophic mice treated with Idebenone (mdxI); and dystrophic mice treated with LEDT and Idebenone (mdxL+I). Scale bar 100 μm, 20x.
(TIF)

S6 Fig Original images: Muscle cell differentiation regulators in quadriceps muscle. (A) Representative membranes stained with Ponceau of the different bands. (B) Representative bands for MHC-fast, MHC-slow, Myo-D, and myogenin in untreated *mdx* muscle cells (mdxC), mdx muscle cells treated with Idebenone (mdxI); mdx muscle cells treated with LEDT (mdxL) and mdx muscle cells treated with Idebenone and LEDT (mdxL+I). The images in Fig 4B represent one animal per group in sequence (n = 6 animals per group).
(TIF)

S7 Fig Original images: Autophagy-mitophagy markers in quadriceps muscle. (A) Representative membranes stained with Ponceau of the different bands. (B) Representative bands for the LC3 (II/I), SQSTM1/p62, Beclin and Parkin in untreated *mdx* muscle cells (mdxC), mdx muscle cells treated with Idebenone (mdxI); mdx muscle cells treated with LEDT (mdxL) and mdx muscle cells treated with Idebenone and LEDT (mdxL+I). The images in Fig 4B represent one animal per group in sequence (n = 6 animals per group).
(TIF)

S8 Fig Original images: Regulation of autophagy/signaling pathways in quadriceps muscle. (A) Representative membranes stained with Ponceau of the different bands. (B) Representative bands for the AMPK, p-AMPK, m-TORC1, and NF-κB in untreated *mdx* muscle cells (mdxC), mdx muscle cells treated with Idebenone (mdxI); mdx muscle cells treated with LEDT (mdxL) and mdx muscle cells treated with Idebenone and LEDT (mdxL+I). The images in Fig 4B represent one animal per group in sequence (n = 6 animals per group).
(TIF)
