# Supplementary figures and images for: LEDT and Idebenone treatment modulate autophagy and improve regenerative capacity in the dystrophic muscle through an AMPK-pathway
Source: PLoS One. 2024 Mar 18;19(3):e0300006. doi: 10.1371/journal.pone.0300006 (PMC10947673; doi:10.1371/journal.pone.0300006)

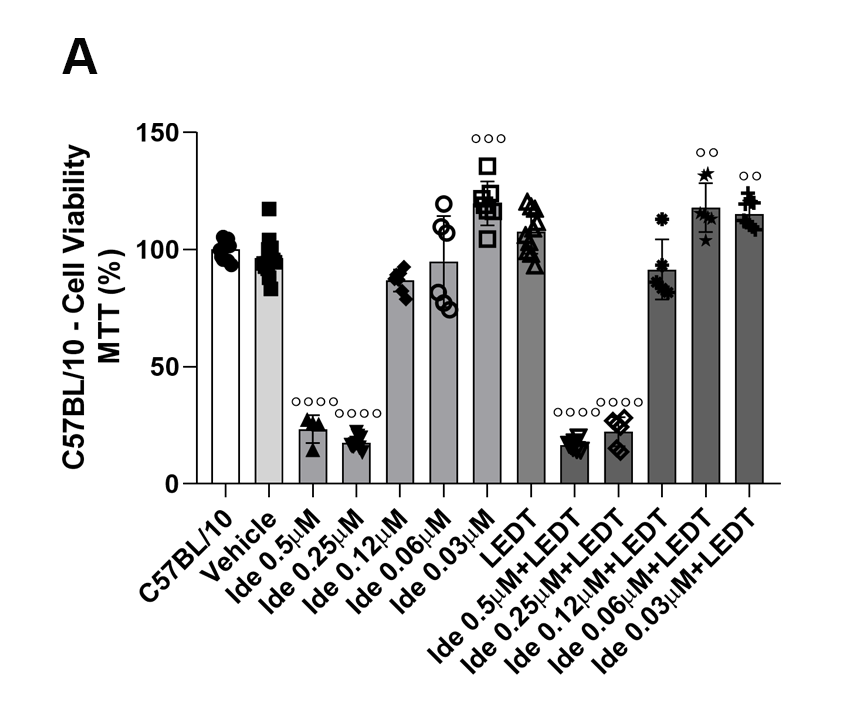

Supplement: S1 Fig — MTT assay in untreated control muscle cells (C57BL/10); control muscle cells treated with carboxymethylcellulose sodium salt (Vehicle); control muscle cells treated with different doses of Idebenone; control muscle cells treated with LEDT (LEDT); and control muscle cells treated with LEDT and different doses of Idebenone, after 48h. All data are expressed by mean±SD and the experiments were carried out in triplicate. *P< 0.05 versus C57BL/10; **P< 0.01 versus C57BL/10; ***P< 0.001 versus C57BL/10; ****P< 0.00001 versus C57BL/10;°P< 0.05 versus Vehicle;°°°° P< 0.00001 versus Vehicle; φP< 0.05 versus Ide 0.03μM. One-way ANOVA followed by Tukey post test was used for statistical analysis. (TIF) [file pone.0300006.s001.tif]

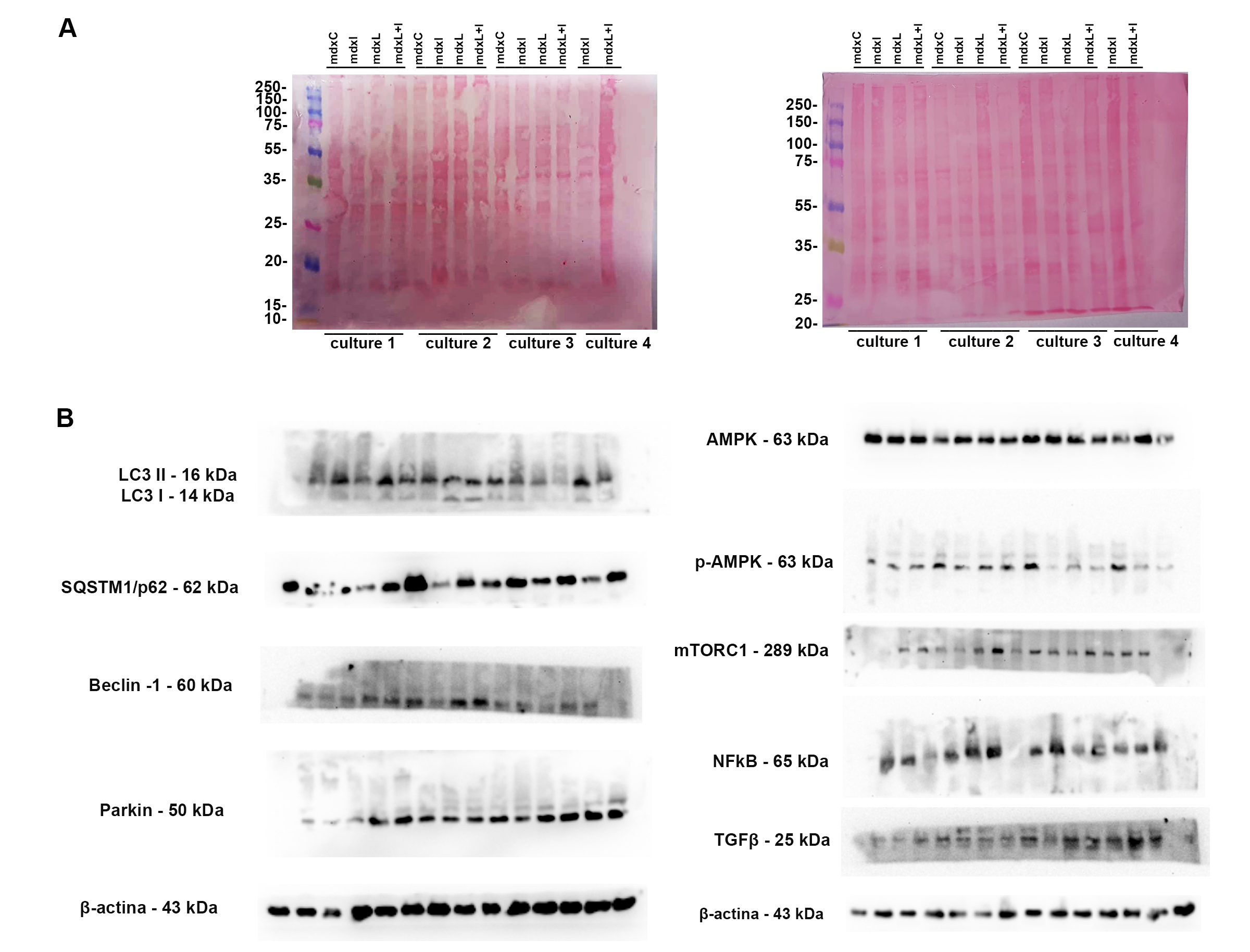

Supplement: S2 Fig — (A) Representative membranes stained with Ponceau of the different bands. (B) Representative bands for the LC3 (II/I), SQSTM1/p62, Beclin, Parkin, AMPK, p-AMPK, m-TORC1, NF-κB and TGF-β in untreated mdx muscle cells (mdxC), mdx muscle cells treated with Idebenone (mdxI); mdx muscle cells treated with LEDT (mdxL) and mdx muscle cells treated with Idebenone and LEDT (mdxL+I). The images in Fig 2B represent 4 independent muscle cell culture per group, in sequence. (TIF) [file pone.0300006.s002.tif]

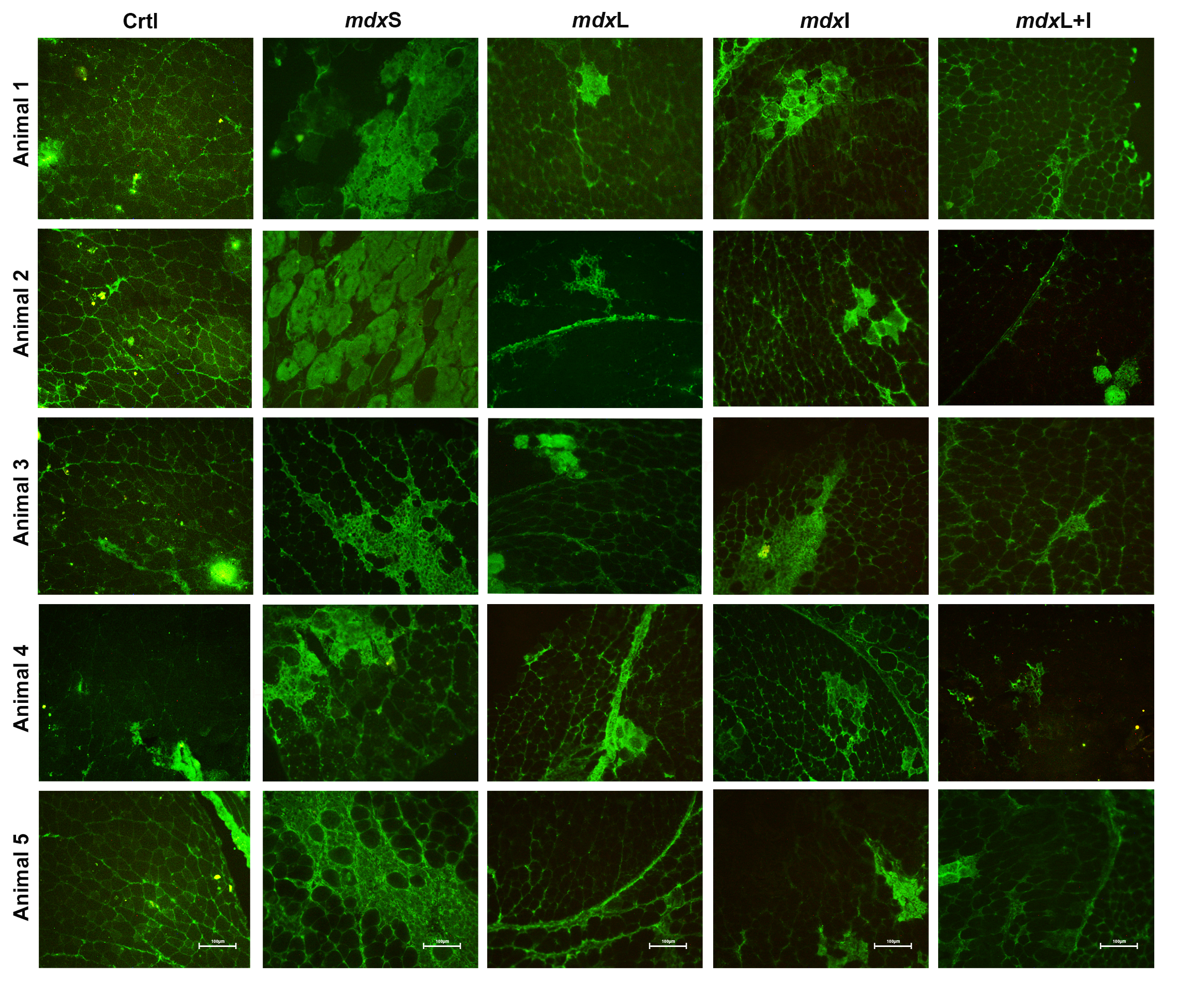

Supplement: S3 Fig — Representative quadriceps cross-sections showing intracellular fiber staining for IgG antibody in the 5 animals from each experimental group: C57BL/10 mice (Ctrl); dystrophic mice received sham LEDT and carboxymethylcellulose sodium salt diluted in water (mdxS); dystrophic mice treated with LEDT (mdxL); dystrophic mice treated with Idebenone (mdxI); and dystrophic mice treated with LEDT and Idebenone (mdxL+I). Scale bar 100 μm, 20x. (TIF) [file pone.0300006.s003.tif]

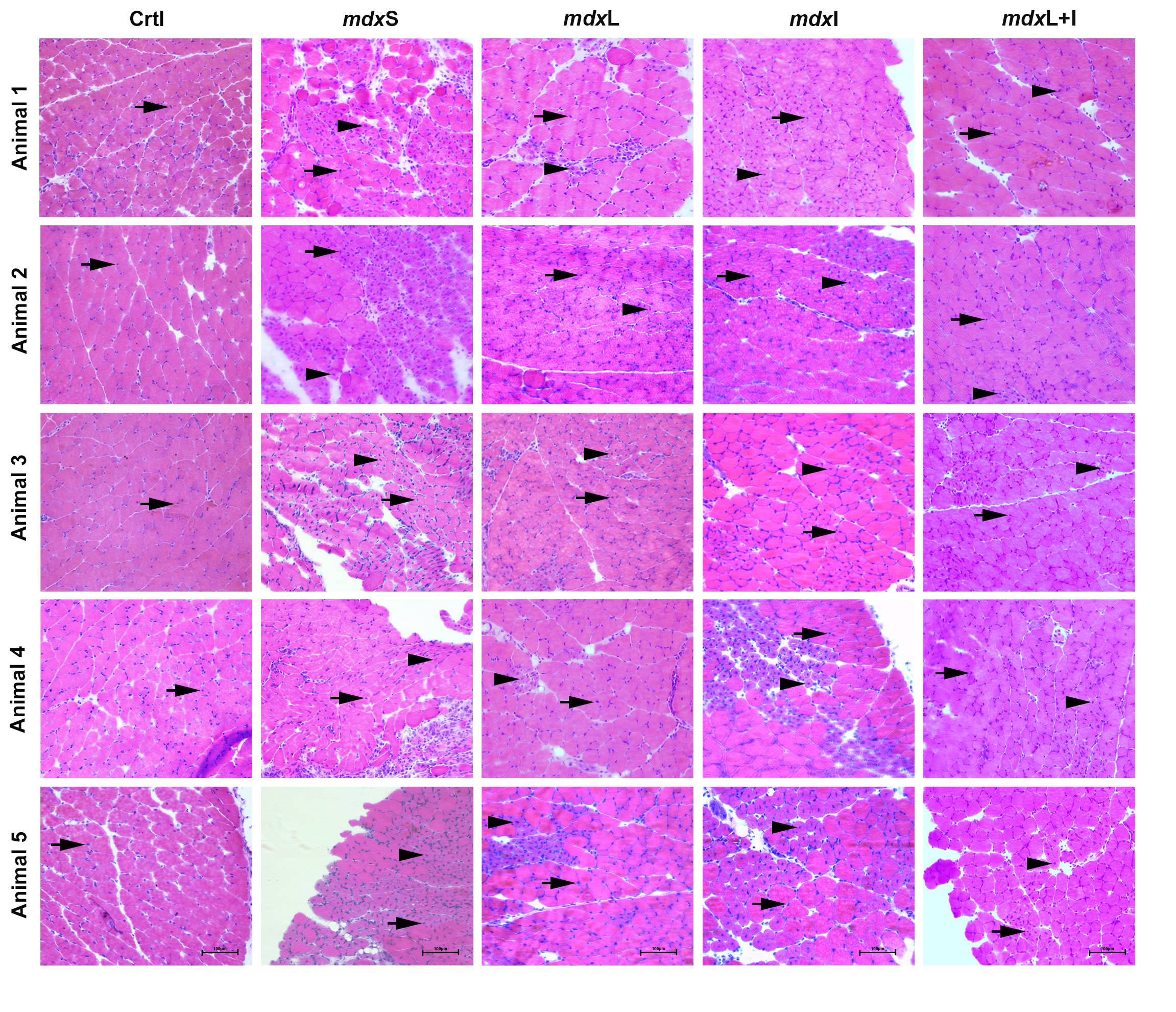

Supplement: S4 Fig — Representative quadriceps cross-sections showing fibers with central nuclei (black arrowheads) and with peripheral nuclei (black heads) in the 5 animals from each experimental group: C57BL/10 mice (Ctrl); dystrophic mice received sham LEDT and carboxymethylcellulose sodium salt diluted in water (mdxS); dystrophic mice treated with LEDT (mdxL); dystrophic mice treated with Idebenone (mdxI); and dystrophic mice treated with LEDT and Idebenone (mdxL+I). Scale bar 100 μm, 20x. (TIF) [file pone.0300006.s004.tif]

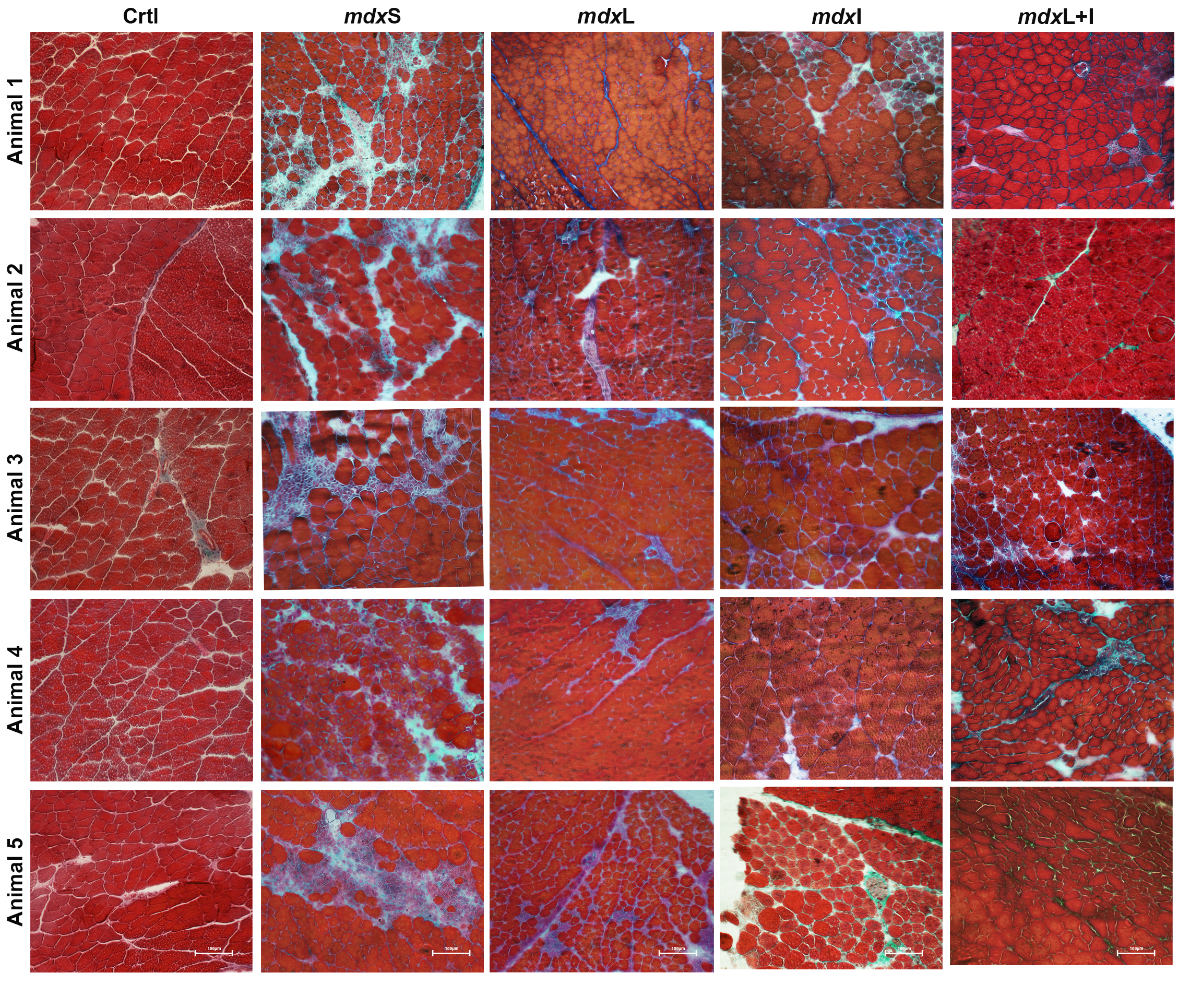

Supplement: S5 Fig — Representative quadriceps cross-sections showing fibrosis area (blue color) in the 5 animals from each experimental group: C57BL/10 mice (Ctrl); dystrophic mice received sham LEDT and carboxymethylcellulose sodium salt diluted in water (mdxS); dystrophic mice treated with LEDT (mdxL); dystrophic mice treated with Idebenone (mdxI); and dystrophic mice treated with LEDT and Idebenone (mdxL+I). Scale bar 100 μm, 20x. (TIF) [file pone.0300006.s005.tif]

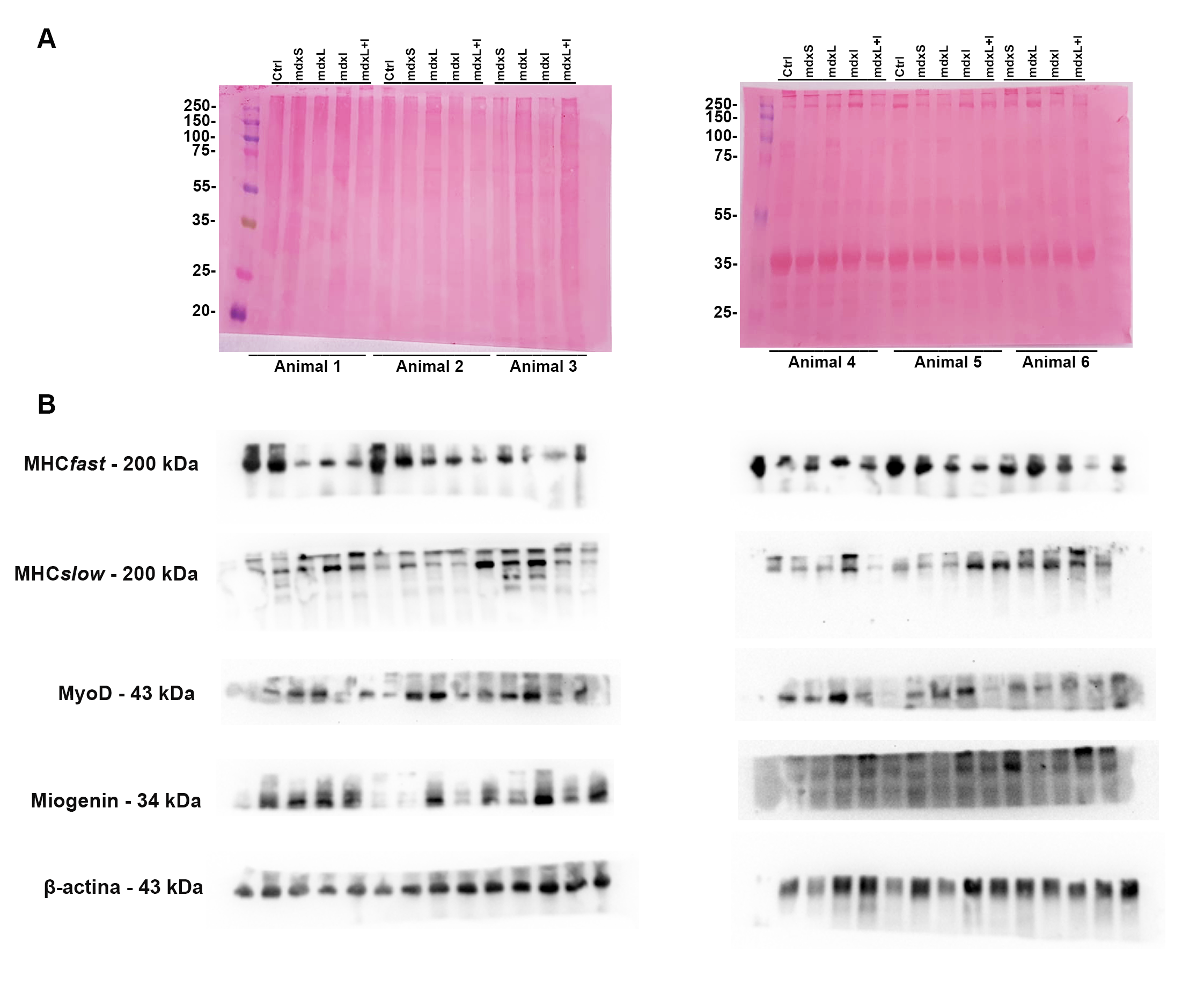

Supplement: S6 Fig — (A) Representative membranes stained with Ponceau of the different bands. (B) Representative bands for MHC-fast, MHC-slow, Myo-D, and myogenin in untreated mdx muscle cells (mdxC), mdx muscle cells treated with Idebenone (mdxI); mdx muscle cells treated with LEDT (mdxL) and mdx muscle cells treated with Idebenone and LEDT (mdxL+I). The images in Fig 4B represent one animal per group in sequence (n = 6 animals per group). (TIF) [file pone.0300006.s006.tif]

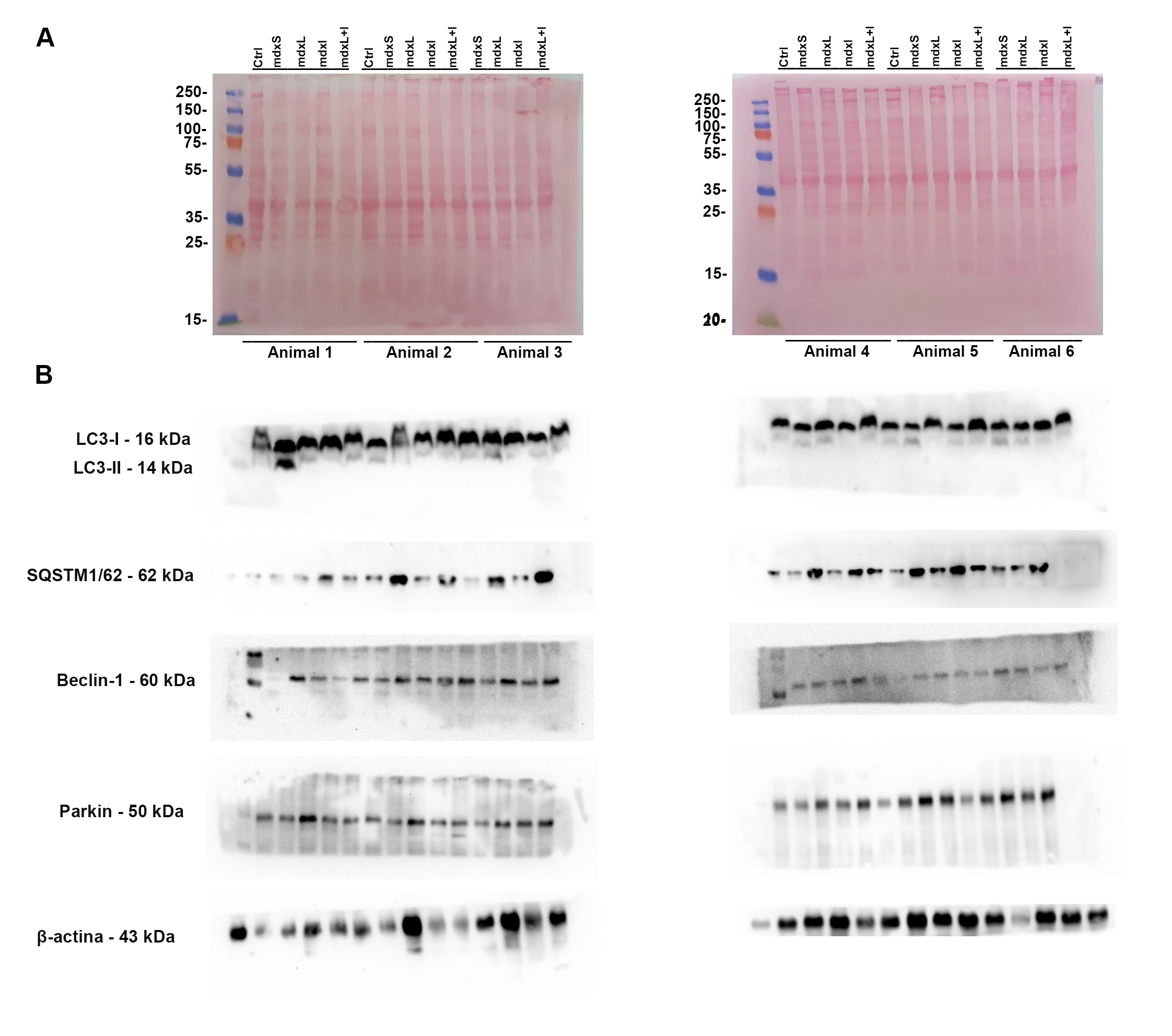

Supplement: S7 Fig — (A) Representative membranes stained with Ponceau of the different bands. (B) Representative bands for the LC3 (II/I), SQSTM1/p62, Beclin and Parkin in untreated mdx muscle cells (mdxC), mdx muscle cells treated with Idebenone (mdxI); mdx muscle cells treated with LEDT (mdxL) and mdx muscle cells treated with Idebenone and LEDT (mdxL+I). The images in Fig 4B represent one animal per group in sequence (n = 6 animals per group). (TIF) [file pone.0300006.s007.tif]

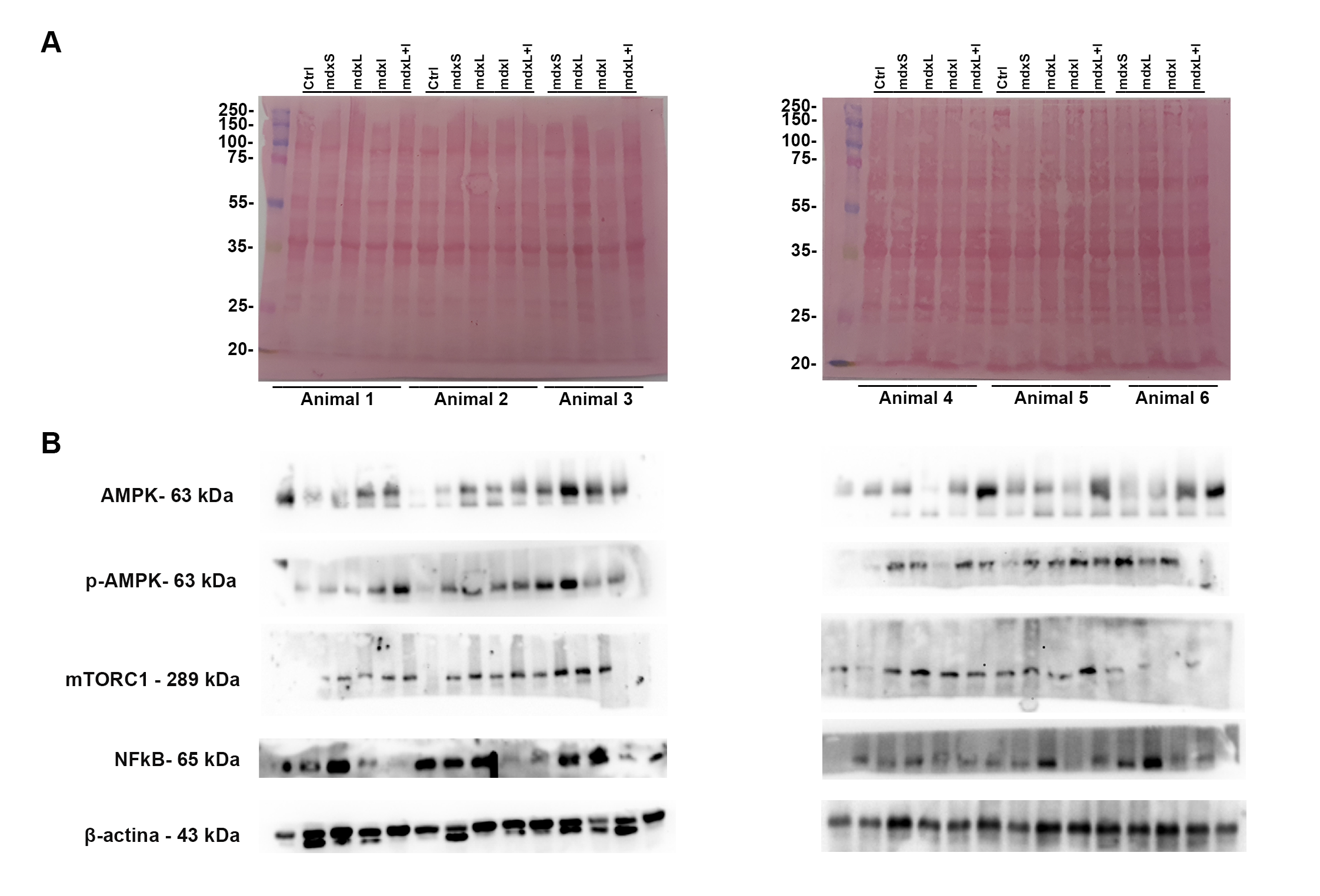

Supplement: S8 Fig — (A) Representative membranes stained with Ponceau of the different bands. (B) Representative bands for the AMPK, p-AMPK, m-TORC1, and NF-κB in untreated mdx muscle cells (mdxC), mdx muscle cells treated with Idebenone (mdxI); mdx muscle cells treated with LEDT (mdxL) and mdx muscle cells treated with Idebenone and LEDT (mdxL+I). The images in Fig 4B represent one animal per group in sequence (n = 6 animals per group). (TIF) [file pone.0300006.s008.tif]
